# Colorectal cancer stem cells develop NK cell resistance via homotypic cell-in-cell structures suppressed by Stathmin1

**DOI:** 10.7150/thno.110379

**Published:** 2025-03-18

**Authors:** Yen-Yu Lin, Hsin-Yi Lan, Hao-Wei Teng, Ya-Pei Wang, Wen-Chun Lin, Wei-Lun Hwang

**Affiliations:** 1 Department of Pathology, Fu Jen Catholic University Hospital, Fu Jen Catholic University, New Taipei City 24352, Taiwan.; 2 School of Medicine, College of Medicine, Fu Jen Catholic University, New Taipei City 24205, Taiwan.; 3 Department of Biotechnology and Laboratory Science in Medicine, National Yang Ming Chiao Tung University, Taipei 112, Taiwan.; 4 Division of Medical Oncology, Department of Oncology, Taipei Veterans General Hospital, Taipei 112, Taiwan.; 5 School of Medicine, National Yang Ming Chiao Tung University, Taipei 112, Taiwan.; 6 Cancer and Immunology Research Center, National Yang Ming Chiao Tung University, Taipei 11221, Taiwan.

**Keywords:** Tumor Microenvironment, Colorectal Cancer Stem Cell, Cell-in-Cell Structure, Immunotherapy, Stathmin1

## Abstract

**Rationale:** Advances in cancer therapies have significantly improved patient survival; however, tumors enriched in cancer stem cells (CSCs) have poor treatment responses. CSCs are a key source of tumor heterogeneity, contributing to therapeutic resistance and unfavorable patient outcomes. In the tumor microenvironment (TME), cell-in-cell (CIC) structures, where one cell engulfs another, have been identified as markers of poor prognosis. Despite their clinical relevance, the mechanisms underlying CIC formation across different tumor cell subpopulations remain largely unknown. Elucidating these processes could provide novel insights and therapeutic opportunities to address aggressive, treatment-resistant cancers.

**Method:** Fluorescent mCherry-carrying colorectal cancer stem cells (CRCSCs) were expanded as spheroids in serum-free media and cocultured with either parental cancer cell-expressing Venus fluorescent protein or CFSE dye-stained immune cells (T cells, M1/M2 macrophages, neutrophils, and NK cells) or treated with EGFR- or PD-L1-targeting antibodies to assess the formation of CIC structures. Genes potentially crucial for the formation of CIC structures were knocked down or overexpressed, and their effects on CIC formation were evaluated. The clinical relevance of the *in vitro* findings was confirmed through analysis of formalin-fixed, paraffin-embedded (FFPE) human colorectal cancer (CRC) specimens.

**Results:** CRCSCs have a strong predilection for serving as the outer cell in a CIC structure and forming homotypic CIC structures predominantly with parental CRC cells. The frequency of CIC structure formation increased when the cells were exposed to anti-PD-L1 antibody treatment. Both the outer CRCSC in a CIC structure and CRCSCs released from a homotypic CIC structure showed enhanced resistance to the cytotoxicity of NK-92MI cells. Restoration of Stathmin1 (STMN1) expression but not *RAC1* knockdown in CRCSCs reduced the homotypic CIC frequency, disrupted the outer cell fate in CIC structures, and increased cell susceptibility to NK-92MI cytotoxicity. In CRC patients, CIC structures are associated with poor tumor differentiation, negative STMN1 expression, and poor prognosis.

**Conclusion:** CSCs play a crucial role in informing CIC structures in CRC. CIC structure formation partially depends on low STMN1 expression and confers a survival advantage under NK cytotoxicity. Targeting this pathway may significantly improve immunotherapy's efficacy for CRC patients.

## Introduction

The tumor microenvironment (TME) consists of a complex assembly of heterogeneous tumor cells alongside various host cells, including fibroblasts, immune cells, endothelial cells, and pericytes. These cells engage in continuous, reciprocal communication to establish a tumor-specific niche that fosters cancer growth and resistance to therapy. Biochemical signaling within the TME arises from several fundamental mechanisms, including spatial ligand-receptor interactions 1, extracellular vesicle (EV)-mediated communication 2, 3, and direct cell-cell contacts 4, 5, all of which dynamically reshape the TME landscape. Ultimately, this interplay within the TME not only drives tumor growth and metastasis but also contributes to the development of treatment resistance and tumor relapse, presenting ongoing challenges for effective cancer therapy 6.

Among the different cell types in the TME, cancer stem cells (CSCs) are crucial, nongenetic drivers of tumor heterogeneity. CSCs have a unique self-renewal capacity and can contribute to metastasis and recurrence 7. CSCs have been discovered in different types of cancers. Several CSC populations have been identified in colorectal cancer (CRC): (1) CD133^+^ CSCs, which have been isolated from primary colorectal tumors 8, 9; (2) ESA^+^/CD44^+^ CSCs, which are increased in xenogeneic colon tumors postchemotherapy 10; (3) CD26^+^ cells, which are enriched from CD133^+^/CD44^+^ populations and are capable of acting as metastasis-initiating CSCs 11; and (4) Lgr5^+^ CSCs, which are essential for the formation and maintenance of liver metastasis 12. In tissue culture of CRC cells, serum-free medium containing bFGF and EGF can enrich cancer stem-like cells, promoting symmetric division. In contrast, cells tend to divide and differentiate asymmetrically in a serum-containing medium 13, 14. Although they express various surface markers, these different populations of CSCs share the standard features of tumor-initiating capability, treatment resistance, and other stem-like properties. Multiple lines of evidence indicate that colorectal cancer stem cells (CRCSCs) can actively orchestrate protumoral TME. CRCSC-produced IL-4 can support tumor growth and cell death resistance 15, while IL-8 can promote endothelial cell migration and tube formation 16. CRCSC-derived small extracellular vesicles (sEVs) can expand immunosuppressive neutrophils 17. Novel mechanisms through which CSCs interact with and modulate the TME are continuously being discovered.

In addition to producing secretory signals and generating tumor cell progenies, CSCs may modulate the TME by forming cell-in-cell (CIC) structures. CIC is a term used in histopathology to describe the phenomenon of a whole cell existing inside another cell 5. CIC structures are evident in cancers, and the presence of CIC structures has been considered an indicator of poor prognosis 18, 19. In homotypic CIC structures, the carcinoma cells are internalized by neighboring carcinoma cells. This phenomenon has been shown to increase the survival of cancer cells; for example, in a chemotherapy-induced senescence model of breast cancer, CIC structures are formed, and the inner cells are broken down, conferring a survival advantage to the outer cells. 20. In contrast, TNF-related apoptosis-inducing ligand (TRAIL), a cytokine capable of inducing cancer cell death, also promotes CIC structure formation, typically resulting in inner cell death 21. In T-cell-mediated anticancer immunity, T-cell-secreted granules also induce the transient formation of homotypic CIC structures between cancer cells; such structures can protect inner cells from T-cell attacks 22. Alternatively, heterotypic CIC structures can be formed by two distinct cell types; these structures can also benefit cancer cells. For example, engulfing mesenchymal stem cells (MSCs) by cancer cells enables survival under low-serum conditions and induces cancer dormancy 23. The heterotypic CIC structure generated by the internalization of NK cells within cancer cells can either cause in-cell NK killing 24 or activate signal transduction pathways, including AKT signaling, and promote cancer cell growth and drug resistance while reducing sensitivity to NK cells 25. The phenotypes of CIC structures, including increased survival and treatment resistance, are similar to those of CSCs. We hypothesize that, compared with nonstem cancer cells, CSCs may be more actively engaged in forming CIC structures and take advantage of the associated fitness benefits. However, little is known about the roles of CSCs in forming CIC structures. Whether the formation of CIC structures is a significant pathway for CSCs to modulate the TME is mainly unaddressed.

To test our hypothesis, in this study, we expanded CRCSCs as cancer spheroids *in vitro* and observed that these cells actively formed CIC structures. We demonstrated that decreased expression of STMN1 was required for homotypic CIC structure generation. The CRCSCs involved in CIC structures exhibited increased NK cell resistance.

## Methods

### Clinical cohort

Patients who were diagnosed with colorectal adenocarcinoma and underwent surgical resection of the primary tumor with curative intent at Fu Jen Catholic University Hospital between 2017 and 2021 were included in this study. The patients were retrospectively identified by reviewing their electronic medical records. We compared patients with poorly differentiated adenocarcinoma cases with patients with well-differentiated to moderately differentiated adenocarcinoma at a 1:1 ratio. This study conforms to the Declaration of Helsinki and was approved by the institutional review board of Fu Jen Catholic University Hospital (FJUH111178). Informed consent was waived, and patient characteristics are shown in **Table S1**.

### Cell culture and expansion of sphere-derived cancer stem cells (SDCSCs)

The human CRC cell lines HCT15 (RRID: CVCL_0292) and HT29 (RRID: CVCL_A8EZ) were cultured in RPMI 1640 medium (Gibco). Human embryonic kidney (HEK) 293 cells (RRID: CVCL_0045) were maintained in DMEM (Gibco). The human leukocyte cell lines HL60 (RRID: CVCL_0002), THP-1 (RRID: CVCL_0006), and Jurkat (RRID: CVCL_0065) were maintained in RPMI-1640 medium with ATCC formulation (Gibco). The above media were supplemented with 10% fetal bovine serum (FBS, Gibco) and 1% penicillin/streptomycin (Gibco). The human natural killer (NK) cell line NK-92MI (RRID: CVCL_3755) was cultured in alpha MEM (Gibco) supplemented with 12.5% FBS (HyClone), 12.5% horse serum (Gibco), 0.02 mM folic acid (Sigma‒Aldrich), 0.1 mM 2-mercaptoethanol (Gibco) and 0.2 mM inositol (Sigma‒Aldrich). The above cells were initially purchased from ATCC. HL60 cells were cultured in 1.25% DMSO (Fisher BioReagents) for 8 days to promote neutrophil differentiation. The cells were then attached to glass slides through a cytospin at 500 rpm for 5 minutes (Cytospin 3, Thermo Shandon) and subjected to Liu's stain (Tonyar biotech. Inc.) according to the manufacturer's protocol. THP-1 cells were treated with 150 nM phorbol 12-myristate 13-acetate (PMA) (Sigma‒Aldrich) for one day and maintained in complete RPMI medium for another day to achieve macrophage differentiation (THP-1-M0). The THP-1-M0 cells were treated with 20 ng/mL IFN-γ (PeproTech) and 10 pg/mL LPS (Sigma‒Aldrich) for one day to induce M1-type macrophage differentiation (THP-1-M1) or 20 ng/mL IL-4 (PeproTech) and 20 ng/mL IL-13 (PeproTech) for three days to induce M2-type macrophage differentiation (THP-1-M2). We utilized a previously defined stem-cell cultivation method to expand and enrich SDCSCs from CRC cell lines 16, 26. Dissociated CRC cells were cultured in DMEM/F12 medium (Gibco) supplemented with N2 Plus Supplement (Gibco), 10 ng/mL bFGF (PeproTech Asia), 10 ng/mL EGF (PeproTech Asia) and MycoExpert (Capricorn Scientific) for 21 days to obtain tumor spheroids. All the cells were cultured under 5% CO_2_ in a humidified incubator (NUAIRE). The authenticity of the cell lines was verified by examining their DNA short tandem repeat (STR) profiles over the previous three years, and all experiments were performed with mycoplasma-free cells.

### Lentivirus production and transduction

HEK293 cells (2x10^6^) were seeded in 10 cm dishes and cultured overnight for virus production. A total of 2.5 μg of VSV-G (National RNAi Core Facility, Academia Sinica); 9 μg of ∆8.9 (National RNAi Core Facility, Academia Sinica); 10 μg of pLenti-STMN1 (OriGene), pLenti-Vector (OriGene) or RAC1 shRNA plasmid (TRCN0000004871 and TRCN0000004873); and 20 μL of T-pro NTR III transfection reagent (T-pro biotechnology) were mixed in 1 mL of basal DMEM for 30 min at room temperature and added dropwise to the cells. To generate mCherry- and Venus-expressing cells, 10 μg of pMDLg/pRRE (Addgene), 5 μg of pRSV-Rev (Addgene), 2 μg of VSV-G, 10 μg of LeGO-V2 (Venus, Addgene) or 10 μg of LeGO-C2 (mCherry, Addgene) were transfected into HEK293 cells. The lentiviral particle supernatant was collected and transduced into the indicated cells with 8 μg/mL polybrene (Sigma‒Aldrich) before cell sorting with a CytoFLEX SRT cell sorter (Beckman Coulter).

### Quantification of CIC structures *in vitro*

To detect the formation of heterotypic CIC structures, 6x10^4^ mCherry-carrying SDCSCs or parental CRC cells were mixed with 6x10^4^ Venus-carrying parental CRC cells or CFSE-stained immune cells and seeded in basal RPMI medium in a 24-well plate precoated with 5 mg/mL poly 2-hydroxyethyl methacrylate (poly-HEMA) (Sigma‒Aldrich) for 24 h. The CIC structures were quantified via an Olympus IX83 inverted microscope (Olympus Corporation) equipped with a humidified cell chamber with 5% CO_2_. An established CIC structure was defined as more than 50% of an inner cell enclosed within an outer cell under 200x magnification. To monitor homotypic CIC structure formation, 7x10^4^ mCherry-carrying SDCSCs were mixed with 7x10^4^ Venus-carrying parental cells in the presence of 2x10^6^ unlabeled immune cells in basal RPMI medium or treated with 200 μg/mL IgG control antibody (Bioxcell), 200 μg/mL cetuximab (Merck), or 200 μg/mL anti-PD-L1 antibody (B7-H1) (Bioxcell) for 48 h. Y27632 (50 μM) (Cell Signaling Technology) was added to induce ROCK inhibition during homotypic CIC structure formation for 24 h. The numbers of CIC structures counted are shown in the corresponding figure legends.

### Time-lapse tracking and confocal imaging

To quantify the cell fate of CIC structures in the presence of NK cell cytotoxicity, 7x10^4^ mCherry-carrying SDCSCs were mixed with 7x10^4^ Venus-carrying parental cells overnight in a 24-well plate. Then, 2x10^6^ NK-92MI cells per well were added, followed by cell tracking with 30-min image intervals under an Olympus IX83 inverted microscope for 24 h (HT29 cells) and 4 h (HCT15 cells). The death of CRC cells was defined as the loss of a fluorescence signal. To image CIC structure formation, 6×10^4^ CRC cells were seeded in basal RPMI medium and cultured in one compartment of a 3.5 cm four-compartment dish (Greiner Bio-One) for 24 h. Time-lapse imaging was performed with a confocal microscope (LSM880, Zeiss) in a humidified cell chamber with 5% CO_2_​. Cell tracking was conducted at 30-minute intervals over an additional 48-hour period.

### RNA extraction and real-time quantitative PCR (RT‒qPCR)

The RNA isolation and complementary DNA (cDNA) preparation protocols were described previously 26. The diluted cDNA was mixed with SYBR Green master mix (Thermo Fisher) and the indicated primer sets. PCR was carried out with a StepOnePlus^TM^ Real-Time PCR System (Applied Biosystems Inc.). The primer sequences are listed in **Table S2**.

### Flow cytometry analysis

A total of 2x10^5^ cells were suspended in 100 μL of FACS buffer containing 1% bovine serum albumin (BSA, Bioshop) and 2 mM ethylenediaminetetraacetic acid (EDTA, J.T. Baker) prepared in cooled 1x phosphate-buffered saline (PBS, Bioman) for hybridization on ice for 30 min with the following primary antibodies: Alexa Fluor 647-conjugated anti-PD-L1 extracellular domain (ECD) (Abcam), Alexa Fluor 647-conjugated anti-EGFR (BioLegend) or Alexa Fluor 647-conjugated IgG control (BioLegend). The cells were washed with FACS buffer and fixed with 2% paraformaldehyde (PFA, Sigma‒Aldrich) at 4 °C for 30 min before analysis with a Beckman Coulter CytoFLEX flow cytometer. The antibodies used are listed in **Table S3**.

### Immunoblotting and RAC1 pull-down assay

The total protein concentration obtained from cultured cells was quantified with a BCA protein assay kit (Thermo Fisher), and lysates were then subjected to electrophoresis or pull-down assays as described previously 27. Briefly, pellets from GST-tagged PAK1 fusion protein-expressing bacteria were resuspended in 1 mL of 1% Triton X-100 (Sigma‒Aldrich) and sonicated with a Qsonica Q700 sonicator. GST-tagged PAK1 was incubated with 100 μL of glutathione (GSH) Sepharose (Cytiva) under a rotator at 4 °C for 1 h. Then, 250 μg of total cell lysate was added to the GST-protein-bound beads at 4 °C for 1 h. The cleaned beads were suspended in 2x Laemmli sample buffer and boiled at 95 °C for 10 min, and the bead-free supernatant was subjected to western blotting. The immunoblots were visualized with an ImageQuant LAS 4000 chemiluminescence detection system (GE Healthcare Bio-Sciences, USA) and quantified with ImageJ software. The antibodies used are listed in **Table S3**, and the uncropped images are shown in **Figure S8**.

### NK cell cytotoxicity assay

To compare the NK cell cytotoxicity of parental cells and SDCSCs, 1x10^4^ cells in 100 μL of basal RPMI medium were seeded per well overnight in 96-well plates and treated with NK-92MI cells prepared in equal volumes of NK cell medium at different effector (E)/target (T) cell ratios for 3 h (HCT15) or 24 h (HT29) before the MTT assay.

To investigate the effect of ROCK inhibition on cancer cell sensitivity to NK killing, 2x10^4^ parental CRC cells in 100 μL of complete RPMI medium were seeded per well overnight in 96-well plates in the presence or absence of Y27632 (50 μM). The medium was washed away, and 1x10^4^ NK-92MI cells were added to 50 μL of NK cell medium and 50 μL of complete RPMI medium for 3 h before the MTT assay.

### Cell viability and spheroid formation assay

Cell viability was assessed using the thiazolyl blue tetrazolium bromide (MTT) (Sigma‒Aldrich). The culture medium was discarded, and a medium containing 5 mg/mL MTT reagent was added to the cells for 45 min. MTT crystals were then dissolved in 100 μL of dimethyl sulfoxide (DMSO, Scharlau), and the absorbance at 560 and 670 nm was measured with a microplate reader (Infinite M200 Pro, Tecan). For the sphere formation assay, 5x10^3^ CRC cells were resuspended in DMEM/F12 medium (Gibco) supplemented with N2 Plus supplement (Gibco), 10 ng/mL bFGF (PeproTech Asia), and 10 ng/mL EGF (PeproTech Asia). The cells were seeded in 96-well plates for 8 days. The spheroids with a diameter of greater than 50 μm were counted.

### Quantification of CIC structures in FFPE sections of human CRC specimens

The recognition criteria for a CIC structure were based on a previous publication 28. One representative formalin-fixed, paraffin-embedded (FFPE), four micrometer-thick, hematoxylin and eosin (HE)-stained section from each patient's colorectal tumor was examined under 400x magnification to quantify CIC structures in ten high-power fields (hpfs). Structures within this examined area that fulfilled at least four of the following six criteria were counted as CIC structures, and the total number was recorded: (1) the nucleus of the internalized cell was visible; (2) the cytoplasm of the internalized cell was visible; (3) the nucleus of the engulfing cell was visible; (4) the cytoplasm of the engulfing cell was visible; (5) the nucleus of the engulfing cell showed a “moon shape” deformity; and (6) a vacuolar space was identified between the internalized cell and the engulfing cell. We counted only structures in which both the internalized and the engulfing cells could be morphologically identified as carcinoma cells, and incomplete structures, such as cancer cell nuclear molding without total internalization, were omitted. Images were evaluated with an Olympus BX43 microscope equipped with a DP22 CCD camera (Olympus).

### Double immunofluorescence staining of CIC structures in FFPE sections

Four micrometer-thick FFPE sections were made from representative tissue blocks from each patient. The sections were dewaxed, rehydrated, and subjected to antigen retrieval in pH 9.0 Tris-EDTA buffer at 95 °C for 20 min via the PT Link platform (Dako, Glostrup, Denmark). The sections were then blocked with 5% goat serum in phosphate-buffered saline with 0.4% Triton X-100 prepared in 5% serum PBST at 20-25 °C for 1 h. The sections were then incubated with primary antibody solution, including mouse anti-human cytokeratin (Agilent) and rabbit anti-human CD45 (Cell Signaling Technology) in 1% serum PBST at 20-25 °C for 1 h. The sections were washed with 1% PBST and then incubated with a secondary antibody solution including Alexa Fluor 488-conjugated goat anti-mouse IgG (Abcam) and Alexa Fluor 546-conjugated goat anti-rabbit IgG (Thermo Fisher Scientific) at 20-25 °C for 1 h. The sections were washed with 1% serum PBST. Coverslips were mounted with a mounting medium containing DAPI (Abcam), and the sections were examined under an Axio Imager. A D2 fluorescence microscope with a 40x objective (Carl Zeiss Microscopy) was used. Ten CIC structures were identified on each slide, and the expression of CK or CD45 by the internalized and engulfing cells was recorded.

### Three-dimensional (3D) imaging of CIC structure in FFPE sections

The methods used were modified from a previously published protocol 29. One hundred and fifty micrometer-thick sections were made from the FFPE tissue blocks of the two cases with the highest number of CIC structures. The sections were first dewaxed and rehydrated. The sections were stained with the 20 μg/mL fluorescent lipophilic tracer DiD (Thermo Fisher Scientific) to mark the cell membrane and with DAPI (Sigma‒Aldrich) to mark the nucleus. The stained sections were then immersed in a clearing reagent (JelloX Biotech Inc.) 30-32 overnight at 20-25 °C to make them optically clear. The sections were sealed with clearing reagent and stored at room temperature before imaging.

We transferred the samples to chambered coverslips and used an FV3000 confocal laser scanning microscope (Olympus) to capture fluorescence images of an area of interest from each case that was 0.8 × 0.8 cm in size in a two-dimensional (2D) area. The location was selected based on previous H&E-stained sections that contained CIC structures. We acquired images with FV31S-SW software (Olympus) at 0.7 μm intervals along the Z axis. After normalization with Imaris 9.7 software (Bitplane, RRID: SCR_007370, Belfast, UK), the 2D images were exported as individual files. We examined the 2D images at the mid-depth level of each case and identified CIC structures based on the same criteria used in traditional HE-stained sections. We then used Imaris software to create 3D images of individual CIC structures.

### Immunohistochemistry (IHC)

FFPE sections of human CRC specimens were deparaffinized and autoclaved in 10 mM citric acid (Honeywell) buffer for antigen retrieval at pH 6.0. The sections were immersed in 3% H_2_O_2_ for 10 min, followed by 0.1% Triton X-100 for 5 min at room temperature. The primary antibody against STMN1 (Cell Signaling Technology) was diluted with antibody dilution buffer (Ventana) and hybridized with the sections at 4 °C overnight. The tissue sections were washed three times and incubated with anti-immunoglobulin cocktails (BioGenex) for 30 min at room temperature and then with streptavidin peroxidase (BioGenex) for 20 min at room temperature. DAB solution (Epredia) was used for visualization. The sections were counterstained with Mayer's hemalum solution (Sigma‒Aldrich) and mounted with Kaiser's glycerol gelatin mounting medium (Millipore). The histology score (H score) was defined as the percentage of the STMN1-positive immunostained region (0 to 100) multiplied by the intensity of STMN1 staining (0, 1, 2, and 3). An H score of more than 100 was considered positive immunoreactivity. Images were evaluated with an Olympus BX43 microscope equipped with a DP22 CCD camera (Olympus). All reagents and chemicals used are summarized in **Table S4**.

### Statistical analysis

SPSS software (version 16.0) or GraphPad Prism (version 9.5.1) was used for the statistical analyses. The normality of the data was checked via the Shapiro-Wilk test. Two-sided Student's t test or ANOVA followed by Tukey's post hoc test was used to compare data groups with a normal distribution. The Mann‒Whitney *U* test was performed when the data did not follow a normal distribution. Fisher's exact test analyzed correlations between clinicopathological variables and STMN1 immunoreactivity. The log-rank (Mantel-Cox) test was used for survival analysis. A p value less than 0.05 was considered statistically significant.

## Results

### CRCSCs generate homotypic CIC structures with parental CRC cells and serve as outer cells in both homotypic and heterotypic CIC structures

To investigate the cell-cell interactions among CRCs, CRCSCs, and host immune cells, we first prepared fluorescence-labeled parental CRCs, CRCSCs, and various leukocytes *in vitro*.

We ectopically expressed Venus or mCherry fluorescent proteins in HT29 and HCT15 CRC cells via lentiviral vectors. The Venus-labeled **(Figure S1A)** and mCherry-labeled CRC cells **(Figure S1B)** were sorted to over 90% purity. The Venus-labeled CRC cells were maintained as parental cells and not further modified. mCherry-labeled CRC cells were cultured in a defined stem-cell medium to produce sphere-derived cancer stem cells (SDCSCs) to approximate the features of CRCSCs **(Figure S1C)**. Compared with mCherry-expressing parental cells, mCherry-expressing SDCSCs presented greater expression of the stemness genes *LGR5* and *CD44* **(Figure S1D)**. The mCherry-expressing SDCSCs also exhibited an enhanced self-renewing ability, as evidenced by their increased capacity to form spheres **(Figure S1E).** These results show that SDCSCs have features of CRCSCs.

We also prepared various leukocyte subsets from multiple cell lines. We cultured the leukemic cell line HL60 under 1.25% DMSO for eight days to promote *in vitro* neutrophil differentiation. Differentiated HL60 cells (dHL60) exhibited a reduced nucleus-to-cytoplasm (N/C) ratio and an increased percentage of cells with segmented nuclei (**Figure S2A-B).** The expression of the neutrophil maturation markers P67PHOX and CYBB 33 was also increased **(Figure S2C)**, indicating that the dHL60 cells presented many features of differentiated neutrophils. We treated the leukemic cell line THP-1 with 150 nM PMA to obtain cells with a macrophage phenotype. The treated THP-1 cells transitioned from a suspension state to an adherent state. These cells were harvested as M0-type macrophages (THP-1-M0) **(Figure S2D)**. These THP-1-M0 cells presented increased expression of the macrophage differentiation markers CD14 and CD36 34-36 **(Figure S2E)**. When the THP-1-M0 cells were further polarized to M1-type macrophages (THP-1-M1) or M2-type macrophages (THP-1-M2) via IFN-γ/LPS or IL-4/IL-13 37, increased expression of *IL-1B* and *TNFA* was observed in the THP-1-M1 cells. In contrast, *CD206* expression was elevated in THP-1-M2 macrophages **(Figure S2F)**. Our protocols generated leukocyte populations with cardinal phenotypes of major human leukocyte subsets. We used Jurkat E6 cells as surrogates for CD4 T cells without further modifications to study lymphocytes.

**Figure 1:**
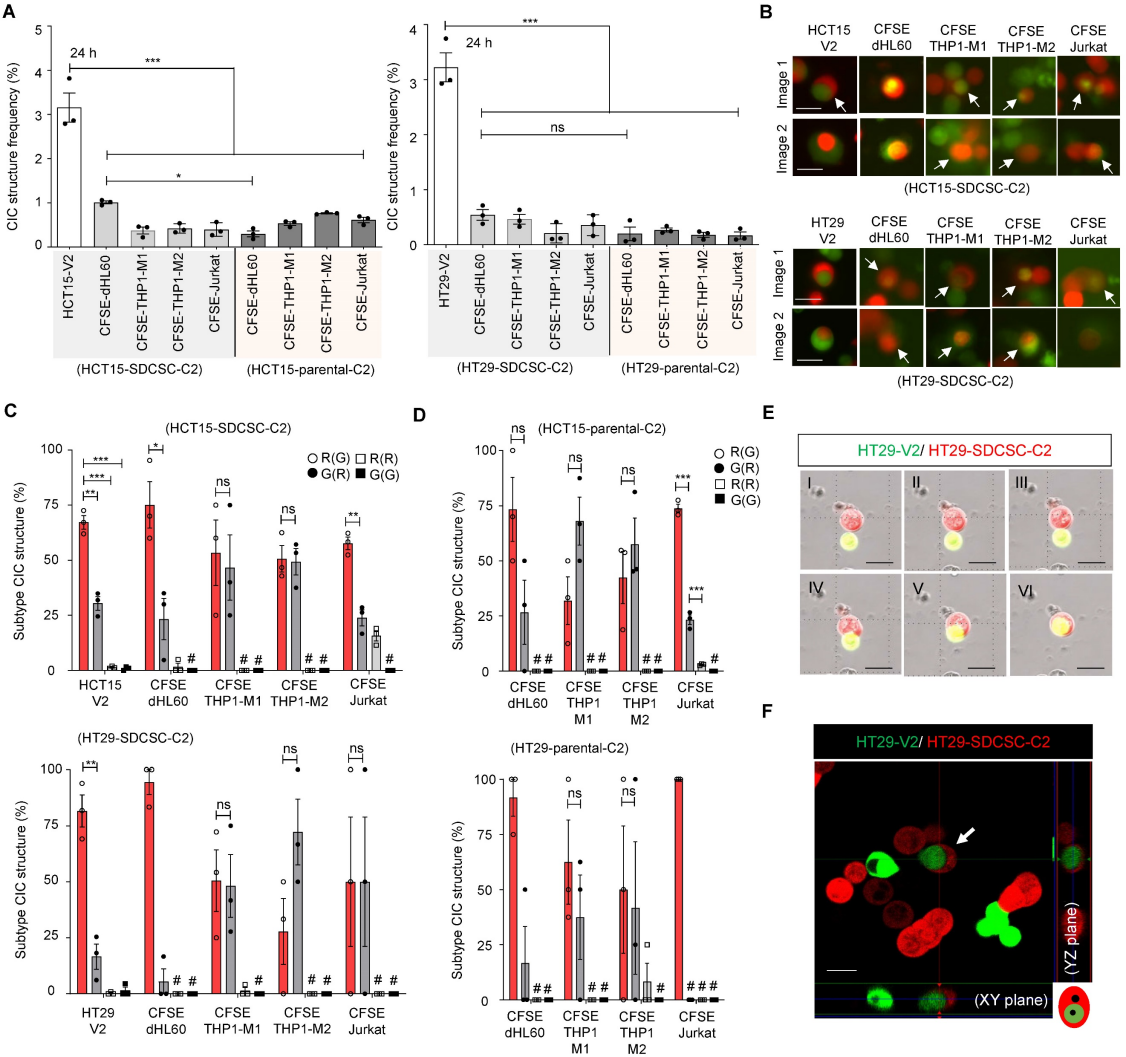
** SDCSCs generate more CIC structures when cocultured with parental CRC cells. (A).** Histograms showing the frequency of CIC structures generated by mCherry-carrying SDCSCs (HCT15-SDCSC-C2 and HT29-SDCSC-C2) and Venus-carrying CRC cells (HCT15-V2 and HT29-V2) or CFSE-stained immune cells 24 h after initial cell seeding in basal RPMI-1640 medium. The data are presented as the means ± sems. *P* < 0*.05, ***P* <* 0.001. *N =* 3.** (B).** Representative images of CIC structure formation by mCherry-carrying SDCSCs (HCT15-SDCSC-C2 and HT29-SDCSC-C2) and Venus-carrying CRC cells (HCT15-V2 and HT29-V2) or CFSE-stained immune cells at 24 h under basal RPMI-1640 medium cultivation. White arrow, CIC structure. Two representative images from three independent assays are shown. Scale bar = 10 μm. **(C).** Percentage of CIC structure subtypes between mCherry-carrying SDCSCs and Venus-carrying CRC cells or CFSE-labeled immune cells. There were 214 (HCT15-V2), 61 (CFSE dHL60), 15 (CFSE THP1-M1), 23 (CFSE THP1-M2), and 39 (Jurkat) CIC structures counted when the indicated cells were cocultured with mCherry-labeled HCT15-SDCSCs. When the indicated cells were cocultured with mCherry-labeled HT29-SDCSCs, 134 (HT29-V2), 17 (CFSE dHL60), 34 (CFSE THP1-M1), 13 (CFSE THP1-M2), and 6 (Jurkat) CIC structures were counted. R(G), CIC structures with outer mCherry-carrying SDCSCs and the inner indicated cells. The data are presented as the means ± sems. ^*^P* <* 0.05, *** P < 0.001; ns, not significant; #, not detected. *N =* 3. **(D).** Percentages of CIC structure subtypes among mCherry-carrying CRC cells (HCT15-C2 or HT29-C2) and CFSE-labeled immune cells. Sixteen (CFSE dHL60), 23 (CFSE THP1-M1), 44 (CFSE THP1-M2), and 57 (Jurkat) CIC structures were counted when the indicated cells were cultured with HCT15-C2 cells. R(G), CIC structures with outer mCherry-carrying CRC cells and inner indicated cells. When the indicated cells were cocultured with mCherry-labeled HT29-C2 cells, 7 (CFSE dHL60), 34 (CFSE THP-1-M1), 13 (CFSE THP-1-M2), and 5 (Jurkat) CIC structures were counted. The data are presented as the means ± sems. *** P < 0.001; ns, not significant; #, not detected. *N =* 3. **(E).** Time-lapse images showing the engulfment of parental HT29-V2 cells by HT29-SDCSC-C2 cells. Scale bar = 10 μm. **(F).** Representative confocal images showing the internalization of an HT29-V2 cell by an HT29-SDCSC-C2 cell—scale bar = 10 μm.

With all the cell populations prepared, we cocultured mCherry-labeled SDCSCs (SDCSC-C2) or mCherry-expressing parental CRC cells (CRC-C2) with CFSE-stained immune cells or Venus-labeled parental CRC cells (CRC-V2) to observe and quantify CIC structure formation. SDCSC-C2 cells formed homotypic CIC structures with CRC-V2 cells, whereas SDCSC-C2 and CRC-C2 cells formed rare heterotypic CIC structures with CFSE-stained immune cells **(Figure 1A-B)**. Strikingly, in more than 70% of the observed homotypic CIC structures formed between SDCSC-C2 cells and CRC-V2 cells, the SDCSC-C2 cells were the outer cells; a greater proportion of outer SDCSC-C2 cells was observed in heterotypic CIC structures between SDCSC-C2 cells and CFSE-stained dHL60 cells **(Figure 1C)**. In contrast, the CIC structures formed between the parental CRC-C2 cells and the leukocyte subsets presented a less consistent inner cell‒outer cell pattern, except that the CRC-C2 cells more frequently served as the outer cells in heterotypic CIC structures with CFSE-labeled Jurkat cells** (Figure 1D)**. To prove the authenticity of the CIC structures, the formation of homotypic CIC structures between HT29-SDCSC-C2 and HT29-V2 cells was monitored through time-lapse imaging **(Figure 1E and Movie S1)**. The internalization of the inner HT29-V2 cells by the outer HT29-SDCSC-C2 cells in a homotypic CIC structure was visualized under a confocal microscope **(Figure 1F)**. These results show that SDCSCs can form homotypic and heterotypic CIC structures *in vitro.* Their behavior differs from parental cancer cells, with a stronger tendency to become the outer cell.

### Anti-PD-L1 treatment promotes homotypic CIC structure formation, and CRCSCs with homotypic structures gain resistance to NK cell-mediated cytotoxicity

Next, we investigated the biological significance of CIC structure formation. We hypothesized that CIC structure formation may be an essential tumor response to stimuli in the microenvironment, specifically to the presence of immune cells or tumor-targeting antibodies. To this end, we monitored changes in the formation of homotypic CIC structures between SDCSCs and parental CRC cells in the presence of immune cells or therapeutic antibodies (anti-EGFR antibody cetuximab or anti-PD-L1).

**Figure 2:**
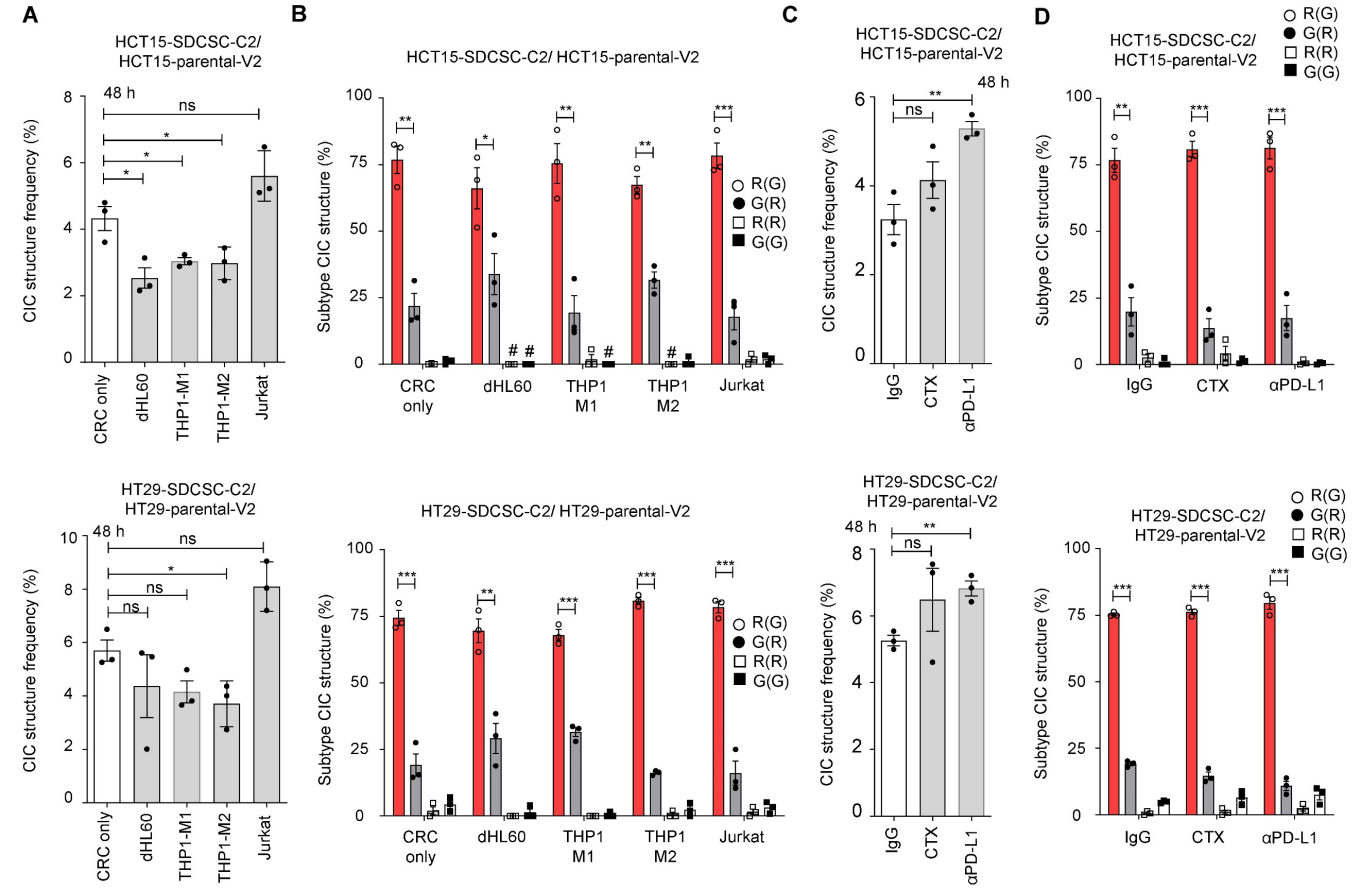
** Increased generation of homotypic CIC structures upon anti-PD-L1 antibody administration. (A).** Histograms showing the frequency of CIC structures 48 h after initial cell seeding in basal RPMI-1640 medium. The data are presented as the means ± sems. *P* <* 0.05; ns, not significant. *N =* 3. **(B).** Percentages of CIC structure subtypes among mCherry-carrying SDCSCs and Venus-carrying CRC cells in the presence of the indicated unlabeled immune cells. There were 400 (no immune cells), 76 (dHL60), 58 (THP1-M1), 96 (THP1-M2), and 329 (Jurkat) CIC structures counted when coculturing HCT15-V2, HCT15-SDCSC-C2, and the indicated immune cells. When HT29-V2, HT29-SDCSC-C2, and the indicated immune cells were cocultured, 361 (no immune cells), 155 (dHL60), 177 (THP1-M1), 151 (THP1-M2), and 538 (Jurkat) CIC structures were counted. R(G), CIC structures with outer mCherry-SDCSCs and inner Venus-CRC cells. The data are presented as the means ± sems. ^*^P* <* 0.05; **P* <* 0.01; ***P* <* 0.001. #, not detected. *N =* 3. **(C).** Histograms showing the frequency of CIC structures after 48 h of culture in the presence of therapeutic antibodies. IgG, IgG control; CTX: cetuximab (Erbitux); αPD-L1, anti-PD-L1 antibody. The data are presented as the means ± sems. **P < 0.01; ns, not significant. *N =* 3. **(D).** Percentages of CIC structure subtypes among mCherry-carrying SDCSCs and Venus-carrying CRC cells in the presence of therapeutic antibodies. A total of 318 (IgG), 328 (CTX), and 467 (αPD-L1) CIC structures were counted when HCT15-V2, HCT15-SDCSC-C2, and the indicated antibodies were cocultured. When HT29-V2, HT29-SDCSC-C2, and the indicated antibodies were cocultured, 412 (IgG), 591 (CTX), and 538 (αPD-L1) CIC structures were counted. R(G), CIC structures with outer mCherry-SDCSCs and inner Venus-CRC cells. The data are presented as the means ± sems. *P < 0.05; **P < 0.01; ***P < 0.001. #, not detected. *N =* 3.

We found that THP1-M2 cells in culture decreased homotypic CIC structures in both HCT15 and HT29 cells **(Figure 2A).** Despite the overall decline, there was no effect on the relative frequency of different types of homotypic CIC structures, with the SDCSC-C2 cell being the outer cell engulfing a parental-V2 inner cell (“R(G)”) remaining the dominant type throughout the different conditions **(Figure 2B)**.

The parental and sphere-derived HCT15 and HT29 cells expressed EGFR on their surface, indicating that they are targetable by the anti-EGFR antibody cetuximab. However, the expression of EGFR on SDCSCs was lower in both cell lines than in the parental cells **(Figure S3A-B).** When the parental cells and the SDCSCs were cocultured in the presence of cetuximab, we detected no change in the frequency of homotypic CIC structure formation **(Figure 2C, middle bars)**. In contrast, in both cell lines studied, the SDCSCs expressed higher levels of PD-L1 than the parental cells **(Figure S3C-D).** When SDCSCs were cocultured with parental cells in the presence of anti-PD-L1, the frequency of homotypic CIC structure formation increased significantly **(Figure 2C, right bars).** In both antibody treatments, the most common type of CIC remains the “R(G)” configuration** (Figure 2D)**.

Because immune checkpoint blockade therapy (ICBT), such as anti-PD-L1 therapy, usually acts through the activation of immune attack on cancer cells 39, we next attempted to recapitulate aspects of the immune reaction *in vitro* and explore the role of homotypic CIC structures in the process. The NK-92MI cell line is a human NK cell line that maintains cytotoxic activity and can kill cancer cells *in vitro* 38. Both HT29 and HCT15 cells are susceptible to NK-92MI-mediated killing, and we found that compared with their parental cells, HT29 and HCT15-SDCSCs presented a modest but significant increase in susceptibility to NK-92MI-induced cytotoxicity in MTT assays **(Figure S4A-B)**.

**Figure 3:**
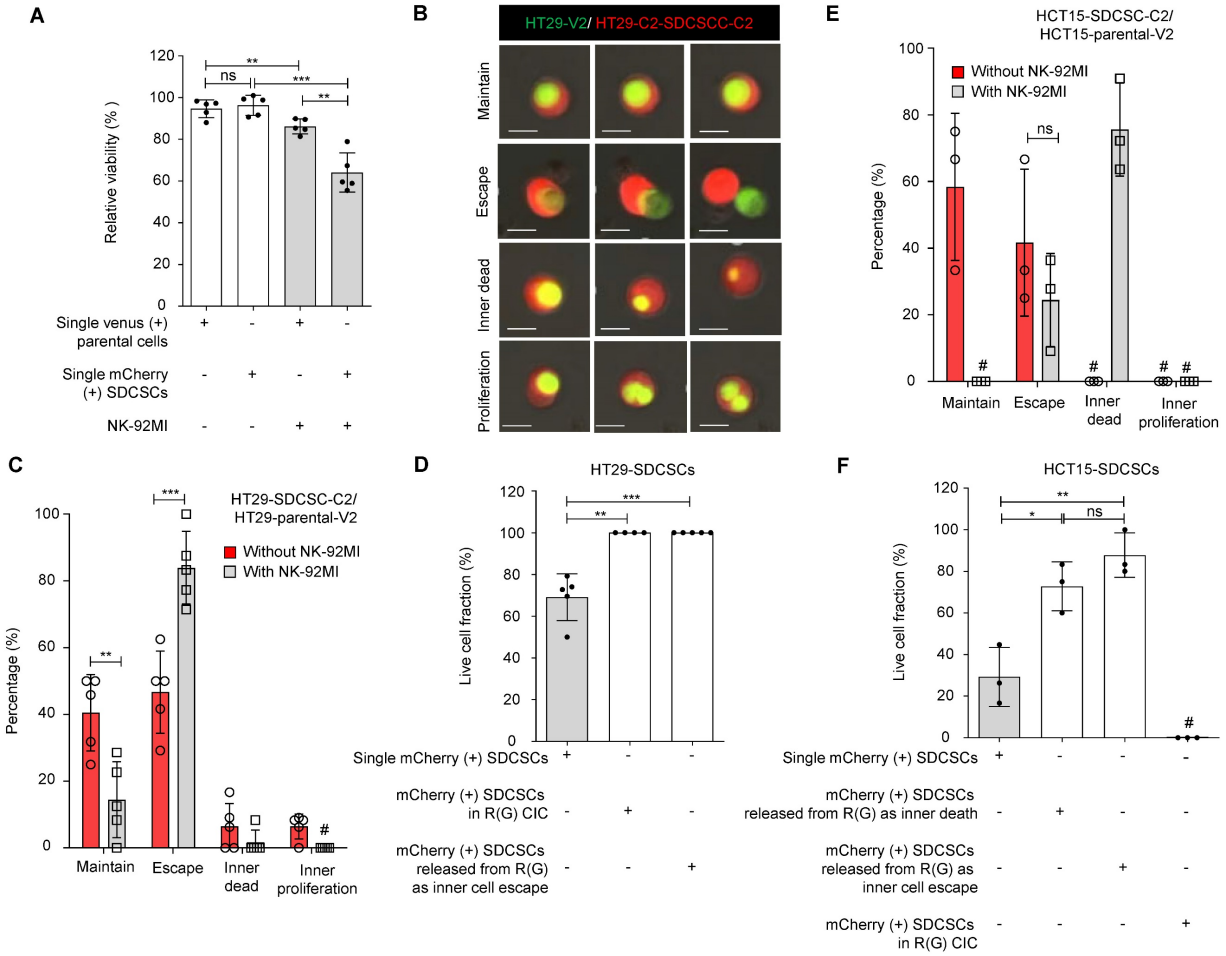
** SDCSCs experiencing a homotypic CIC structure resist NK-92MI cytotoxicity. (A).** Histogram showing the relative viability of the indicated CRC cells. Loss of the fluorescence signal was considered cell death at the end of time-lapse imaging. The data are presented as the means ± sds. **P < 0.01, ***P < 0.001; ns, not significant. *N =* 5. **(B).** Representative time-lapse images of the fates of CIC structures formed by mCherry-expressing HT29-SDCSCs and Venus-labeled HT29 cells. Scale bar = 10 μm. **(C).** A histogram showing CIC structure cell fate in the presence or absence of NK-92MI cells. The data are presented as the means ± sems. **P* <* 0.01; ***P* <* 0.001. #, not detected. *N =* 5. The CIC structure is unchanged through time-lapse imaging; Escape, the release of inner cells; Inner dead, the inner cell is dead in a CIC structure; Inner proliferation, the inner cell is divided within a CIC structure. **(D).** Histogram showing the relative viability of the indicated mCherry-labeled HT29-SDCSCs in the presence of NK-92MI treatment. Cells that lived longer than the average survival time of singlet HT29-SDCSC-C2 cells in the presence of NK-92MI treatment during time-lapse imaging were considered alive, and loss of the fluorescence signal was considered cell death. In total, 105 (single HT29-SDCSC-C2), 10 (outer HT29-SDCSC-C2 cells in a CIC structure), and 42 (single HT29-SDCSC-C2 cells released from R(G) CIC structures) cells were analyzed. The data are presented as the means ± sds. **P < 0.01, ***P < 0.001. *N =* 4-5, as indicated by the number of dots in the histogram. **(E).** The histogram shows the CIC structure cell fate of HCT15 cells in the presence or absence of NK-92MI cells. The data are presented as the means ± sems. ns, not significant; #, not detected. *N =* 3. The CIC structure is unchanged through time-lapse imaging; Escape, the release of inner cells; Inner dead, the inner cell is dead in a CIC structure; Inner proliferation, the inner cell is divided within a CIC structure. **(F).** Histogram showing the relative viability of the indicated mCherry-labeled HCT15-SDCSCs in the presence of NK-92MI treatment. Cells that lived longer than the average survival time of singlet HCT15-SDCSC-C2 cells in the presence of NK-92MI treatment during time-lapse imaging were considered alive, and loss of the fluorescence signal was considered cell death. In total, 204 (single HCT15-SDCSC-C2), 0 (outer HCT15-SDCSC-C2 in a CIC structure), 28 (single HCT15-SDCSC-C2 cells released from R(G) CIC structures due to inner cell escape), and 12 (single HCT15-SDCSC-C2 cells released from R(G) CIC structures due to inner cell death) cells were analyzed. The data are presented as the means ± sds. **P < 0.01; ns, not significant; #, not detected. *N =* 3.

Since HT29 cells are less sensitive to NK-92MI killing, we cocultured HT29-SDCSC-C2 and HT29-V2 cells for one day. Then, we treated them with unlabeled NK-92MI cells at a high E/T ratio to monitor the cell fate within homotypic CIC structures via time-lapse imaging. As expected, compared with parental cells, HT29-SDCSC-C2 cells were more susceptible to NK-92MI-induced death **(Figure 3A)**. Through time-lapse imaging, we found that in an R(G) CIC structure, the inner HT29-V2 cells may either maintain the structure **(Figure 3B and Movie S2)**, escape from the structure **(Figure 3B and Movie S3)**, die within the structure **(Figure 3B and Movie S4)** or proliferate within the structure **(Figure 3B and Movie S5)**. In the presence of NK-92MI cells, the percentage of cells that escaped from the HT29-SDCSC-derived R(G) CIC structure significantly increased **(Figure 3C, escape group)**. Interestingly, although, in separate cultures, the HT29-SDCSC-C2 cells were more susceptible to NK-92MI-mediated killing than parental-V2 cells were, in the HT29-SDCSC-C2/parental-V2 coculture setting, the viability of the HT-29-SDCSC-C2 cells in the R(G) CIC structures was greater than that of the single HT29-SDCSC-C2 cells **(Figure 3D)**. Even those HT-29-SDCSC-C2 cells whose inner HT-29-V2 cells had already escaped maintained the viability advantage** (Figure 3D).** In the HCT15-SDCSC-C2/parental-V2 coculture setting, decreased maintenance of HCT15-SDCSC-derived R(G) CIC structures and increased death of inner parental HCT15-V2 cells in CIC structures were observed in the presence of NK-92-MI cells **(Figure 3E)**. The HCT15-SDCSC-C2 cells whose inner HCT15-V2 cells were dead or had escaped exhibited greater viability than the single HCT15-SDCSC-C2 cells did **(Figure 3F)**. These findings suggest that involvement in a CIC structure may reprogram SDCSCs and enhance their resistance to NK cell killing.

### STMN1 overexpression, but not RAC1 silencing, suppresses homotypic CIC structure formation

**Figure 4:**
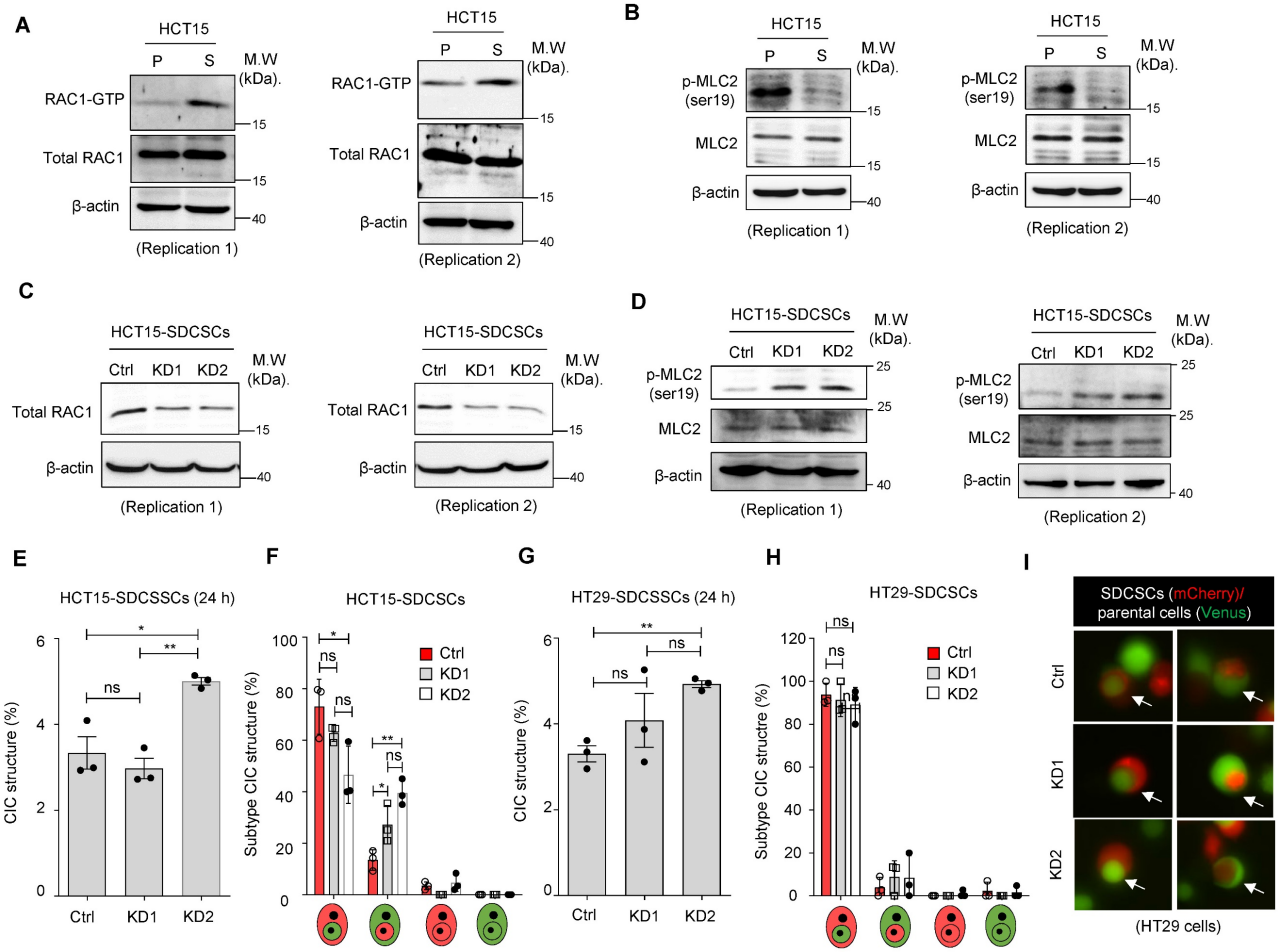
** Knocking down *RAC1* in SDCSCs does not suppress CIC frequency or outer cell fate. (A).** Representative images showing RAC1-GTP and total RAC1 expression in parental cells (P) and SDCSCs (S). M.W., molecular weight. **(B).** Representative images showing phosphorylated MLC2 (Ser19) and total MLC2 expression in parental cells (P) and SDCSCs (S). **(C).** Representative images showing total RAC1 expression in HCT15-SDCSCs receiving scrambled control shRNA (Ctrl) or shRNAs targeting *RAC1* (KD1 and KD2). **(D).** Representative images showing phosphorylated MLC2 (Ser19) and total MLC2 expression in the indicated cells. **(E).** Frequency of CIC structures generated by Venus-labeled parental cells and the indicated mCherry-marked HCT15-SDCSCs (pLKO.1 control and two RAC1-silenced SDCSCs, i.e., KD1 and KD2) in RPMI basal medium for 24 h. Data are presented as the means ± sems. *P < 0.05, ***P < 0.001. ns, nonsignificant. *N =* 3. **(F).** Percentages of inner/outer cell fates of HCT15-SDCSCs carrying pLKO.1 control or shRNAs targeting RAC1 (KD1 and KD2) 24 h after cell seeding. In total, 90 (pLKO.1), 119 (KD1), and 129 (KD2) CIC structures were counted when coculturing HCT15 parental cells (V2) and the indicated HCT15-SDCSCs (C2). The data are presented as the means ± sems. *P < 0.05, **P < 0.01. ns, nonsignificant. *N =* 3. (**G).** The frequency of CIC structures generated by Venus-labeled parental cells indicated that mCherry-marked HT29-SDCSCs were present in the RPMI basal medium for 24 h. Data are presented as the means ± sems. **P < 0.01. ns, nonsignificant. *N =* 3. **(H).** Percentage of inner/outer cell fates of HT29-SDCSCs carrying pLKO.1 control or shRNAs targeting RAC1 24 h after cell seeding. In total, 59 (pLKO.1), 53 (KD1), and 72 (KD2) CIC structures were counted when HT29 parental cells (V2) and the indicated HT29-SDCSCs (C2) were cocultured. The data are presented as the means ± sems. ns, nonsignificant. *N =* 3. **(I).** Representative images of CIC structure formation by mCherry-carrying HT29-SDCSCs receiving pLKO.1 control or shRNAs targeting RAC1 and Venus-labeled CRC cells after 24 h of cultivation in basal RPMI-1640 medium. White arrow, CIC structure. Two representative images from three independent assays are shown. Scale bar = 10 μm.

Next, we investigated the molecular mechanism behind the propensity of SDCSCs to become the outer cells in CIC structures. RAC1, a member of the Rho family of GTPases, is well known to be involved in forming CIC structures, and its activation is associated with outer cell fate 39. We explored the role of RAC1 in forming CICs between SDCSCs and parental cancer cells through a RAC1-GTP pull-down assay. We found that active RAC1-GTP was abundant in HCT15-SDCSCs **(Figure 4A)** and HT29-SDCSC**s (Figure S5A)**. The activation of RAC1 signaling is known to counter RHO/ROCK signaling and decrease MLC2 phosphorylation 40. In our experiments, we also observed reduced levels of phosphorylated MLC2 (Ser19) in HCT15-SDCSCs **(Figure 4B)** and HT29-SDCSC**s (Figure S5B)**. When we knocked down *RAC1* expression in HCT15-SDCSCs **(Figure 4C)** and HT29-SDCSCs **(Figure S5C)**, we observed increased levels of phosphorylated MLC2 (Ser19), as expected **(Figure 4D and Figure S5D)**. However, the knockdown of *RAC1* in SDCSCs had no consistent effect on the frequency of homotypic CIC structure formation in the cocultures of HCT15-SDCSCs and HCT15-parental cells 24 h **(Figure 4E)** or 48 h **(Figure S5E)** after initial seeding. Neither did the knockdown consistently alter the inner-outer cell fate distribution in the CIC structures; the most prevalent CIC composition was still *RAC1*-silenced HCT15-SDCSCs serving as the outer cell and HCT15-parental cells serving as the inner cells **(Figure 4F and Figure S5F)**. After initial seeding, HT29 cell line experiments generated similar results at 24 h **(Figure 4G-I)** and 48 h **(Figure S5G-H)**. These results indicate that increased RAC1-GTP in SDCSCs may not be the main driving force of homotypic CIC structure formation.

**Figure 5:**
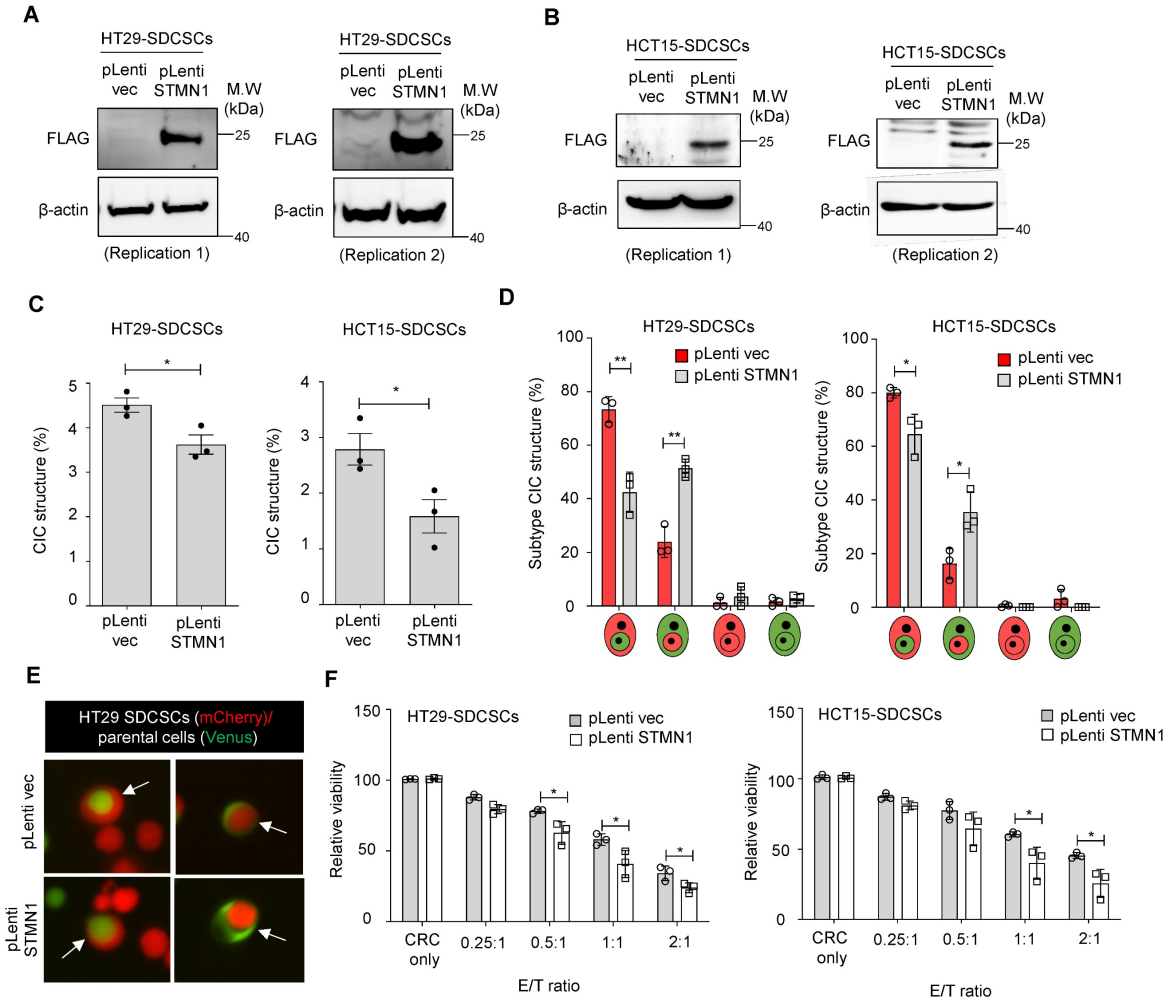
** Overexpression of STMN1 in SDCSCs reduces CIC structure frequency and outer cell fate. (A-B).** Western blots confirm the expression of flag-tagged STMN1 in HT29-SDCSCs (A) and HCT15-SDCSCs (B). **(C).** The frequency of CIC structures in the coculture of Venus-labeled parental cells and vector-treated or STMN1-overexpressing mCherry-marked SDCSCs (in RPMI basal medium for 24 h). The data are presented as the means ± sems. *P < 0.05. *N =* 3. **(D).** Percentages of inner/outer cell fates of SDSCCs carrying pLenti vector or overexpressing STMN1 24 h after cell seeding. In total, 176 (pLenti vec) and 150 (pLenti STMN1) CIC structures were counted when coculturing HT29 parental cells (V2) and the indicated HT29-SDCSCs (C2). A total of 294 (pLenti vec) and 125 (pLenti STMN1) CIC structures were counted when HCT15 parental cells (V2) and the indicated HCT15-SDCSCs (C2) were cocultured. The data are presented as the means ± sems. *P < 0.05, **P < 0.01. *N =* 3. **(E).** Representative images of CIC structure formation by mCherry-carrying HT29 SDCSCs and Venus-labeled HT29 CRC cells at 24 h under basal RPMI-1640 medium cultivation. White arrow, CIC structure. Two representative images from three independent assays are shown. **(F).** The viability of SDCSCs carrying pLenti vector or overexpressing STMN1 upon coculture with NK-92MI cells for 24 h (HT29-SDCSCs) or 3 h (HCT15-SDCSCs) at the indicated effector (E): target cell (T) ratios. The data are presented as the means ± sds. *P < 0.05. *N =* 3.

Another possible driving force of CIC structure formation is Stathmin1 (STMN1), a cytoskeleton regulatory protein. We previously demonstrated that the expression level of STMN1 is lower in SDCSCs than in parental CRCs and that this lower expression maintains their cytosolic softness and contributes to their local invasiveness 41. This increased deformability may be required for CIC structure formation in SDCSCs. Here, we overexpressed Myc-DDK-tagged STMN1 in SDCSCs using a lentiviral vector. STMN1 protein overexpression in HT29-SDCSCs **(Figure 5A)** and HCT15-SDCSCs** (Figure 5B)** was demonstrated via western blotting with an anti-FLAG antibody. Then, we cocultured mCherry-carrying SDCSC-control cells (pLenti-vec) or SDCSC-STMN1-overexpressing cells (STMN1-OE) with Venus-labeled parental CRC cells for 24 h. Overexpression of STMN1 reduced the frequency of homotypic CIC structures **(Figure 5C)** and reduced the proportion of CIC structures in which SDCSCs serve as the outer cells; instead, the proportion of CIC structures with the reverse configuration, i.e., SDCSCs serving as the inner cells wrapped around parental CRC cells, increased **(Figure 5D-E)**. Moreover, STMN1-overexpressing SDCSCs were more susceptible to NK-92MI cytotoxicity **(Figure 5F)**. Our findings indicate that the lower expression level of STMN1 in SDCSCs is an essential molecular feature contributing to their formation of CIC structures and plays a role in SDCSC resistance to immune cell killing.

### STMN1 expression negatively correlates with CIC structure and poorly differentiated CRC characteristics in CRC patients

**Figure 6:**
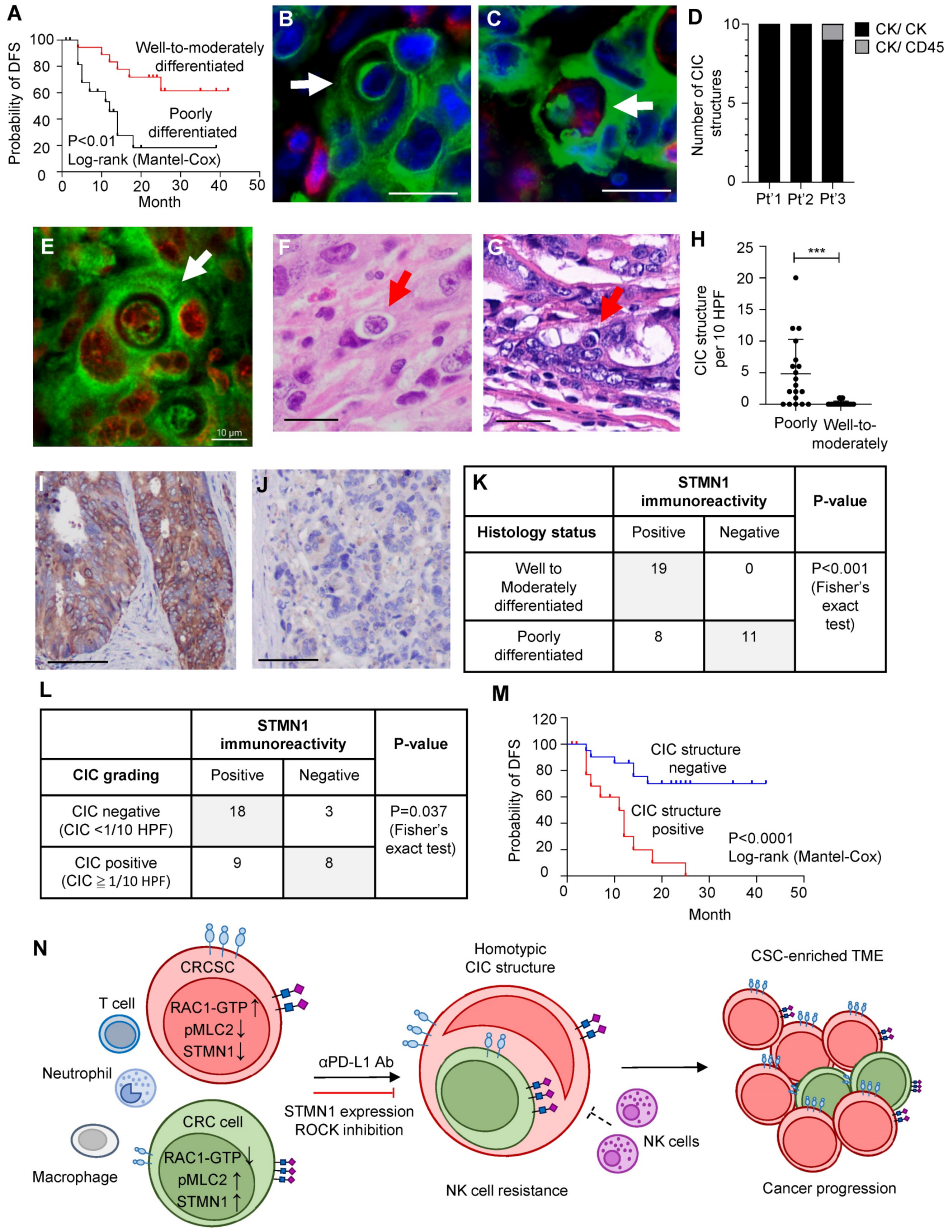
** CIC structures in human colorectal cancer specimens were correlated with poor tumor differentiation, low STMN1 expression, and inferior prognosis. (A).** The Kaplan‒Meier plot shows the probability of disease-free survival (DFS) in the indicated CRC patients. **(B).** Double immunofluorescence revealed that most CIC structures are formed by cytokeratin (CK, green)-positive carcinoma cells as inner and outer cells. Scale bar = 20 μm. **(C).** The image shows only one CIC structure containing a CD45 (red)-positive hematopoietic cell found in three cases. Scale bar = 20 μm. **(D).** CIC structures formed between CK-positive inner and outer cells represent 29 of the 30 CIC structures observed in three cases.** (E).** A single-plane image from three-dimensional imaging indicates a genuine cell-within-cell structure. Scale bar = 10 μm. **(F).** A representative image shows the CIC structure in poorly differentiated adenocarcinoma. Scale bar = 20 μm. **(G).** The image shows the CIC structure in well- to moderately differentiated cases. Scale bar = 20 μm.** (H).** The histogram shows the CIC structure counting in CRC samples. The data are presented as the means ± sds. ***P < 0.001.** (I).** A representative immunohistochemistry image shows a moderately differentiated case that strongly expresses the STMN1 protein. Scale bar = 100 μm. **(J).** A poorly differentiated case is STMN1 negative. Scale bar = 100 μm. **(K).** The poorly differentiated cases were more likely to be STMN1 negative. **(L).** Patients with at least 1 CIC per 10 hpf are more likely to be STMN1 negative than those with less than 1 CIC per 10 hpf. hpf, high-power field. **(M).** The Kaplan‒Meier plot shows the DFS of CRC patients with at least 1 CIC per 10 hpf (CIC structure positive) and those with less than 1 CIC (CIC structure negative). **(N).** Schematic model illustrating the mechanism of CRCSC-associated homotypic CIC structure formation and its role in cancer progression.

To investigate whether our *in vitro* findings can be observed in human patients, we investigated FFPE tumor specimens from 38 colorectal adenocarcinoma patients who had received curative-intent surgeries. Our preliminary observations revealed that CIC structures were more frequently found in poorly differentiated tumors (data not shown); therefore, we included 19 well-to-moderately differentiated patients and 19 poorly differentiated patients in the present study. The patients' clinical and pathological information is summarized in **Table S1**. As expected, patients with poorly differentiated CRC exhibited worse disease-free survival (DFS) than those with well-differentiated to moderately differentiated tumors **(Figure 6A)**.

Next, we observed CIC structures in these human tumor specimens via fluorescence imaging and H&E staining **(Figure 6B-G)**. To further ascertain the identity (epithelial cells vs. leukocytes) of the engulfing cells and the internalized cells in CIC structures, we performed double immunofluorescence studies on the three cases with the greatest number of CIC structures. Double immunofluorescence imaging confirmed that most CIC structures, identified based on morphology features, formed between two cytokeratin (CK)-positive carcinoma cells **(Figure 6B)**. Among the 30 CIC structures from three representative cases examined, 29 were CICs between carcinoma cells, and we found only one CIC structure that was formed by one outer CK-positive carcinoma cell and contained one CK-positive carcinoma cell and one CD45-positive immune cell** (Figure 6C-D)**. To prove that CIC structures identified in four micrometer-thick FFPE sections represent complete CIC structures in 3D space, we utilized tissue clearing technology and 3D imaging methods to observe the entire CIC structure. We found that the inner cells are truly engulfed by the outer cells **(Figure 6E and Movie S6).**

CIC structures can readily be observed in poorly differentiated tumors **(Figure 6F)**. In contrast, CIC structures can rarely be found in well- to moderately differentiated tumors **(Figure 6G)**. The CIC difference between poorly differentiated and well- to moderately differentiated cases was statistically significant **(Figure 6H)**. The average number of CIC structures per 10 hpfs was 4.8 among poorly differentiated cases (standard deviation (SD): 5.4) and 0.2 among well- to moderately-differentiated cases (SD: 0.4). Among the poorly differentiated cases, those with pleomorphic tumor cell morphology **(Figure S6A)** had the highest CIC structure count (mean 9.38, SD 5.41); the count was much lower in poorly differentiated cases with mismatch repair deficiency (MMR-D) (**Figure S6B,** mean 2.28, SD 2.36) and lowest in cases with signet ring cell morphology (**Figure S6C**, mean 0.2, SD 0.43). The differences among these groups were statistically significant (**Figure S6D**).

We next investigated whether STMN1 expression also correlates with CIC structure formation *in vivo*. We demonstrated STMN1 expression in patient tumor specimens via immunohistochemistry (IHC). We noted high **(Figure 6I)** and low **(Figure 6J)** STMN1 immunoreactivity tumors. Using an H score of 100 as the cutoff for distinguishing between STMN1-positive and -negative tumors, we found that the expression of STMN1 was negatively correlated with differentiation status, i.e., negative STMN1 immunoreactivity was more commonly observed in poorly differentiated tumors **(Figure 6K)**. Additionally, positive STMN1 immunoreactivity was correlated with a low CIC frequency in these specimens **(Figure 6L)**. Patients with at least one CIC structure per 10 hpfs (CIC structure positive) had worse disease-free survival than CIC structure-negative patients (**Figure 6M**), demonstrating their prognostic value.

In summary, studies of human samples revealed a correlation between low STMN1 expression and more frequent CIC structure formation, which is compatible with our *in vitro* findings, and this condition is associated with poorly differentiated adenocarcinoma and worse patient survival.

## Discussion

Homotypic CIC structures, typically observed among cancer cells, are often identified in malignant body fluids, including ascites and pleural effusion 42, 43. The presence of CIC structures in solid tumors is associated with poor cancer prognosis 44-46. The formation of CIC structures can be regulated by environmental cues 47 and increased upon irradiation and chemotherapy 48. However, between the seemingly identical inner and outer carcinoma cells of a homotypic CIC structure, the cancer cell subpopulations dictating the distinct outer/inner-cell fates and their corresponding biological significance remain unclear. Our study demonstrated that CRCSCs, a fundamental source of tumor heterogeneity, rarely form heterotypic CIC structures with immune cells but predominantly serve as the outer cells in homotypic CIC structures with CRC cells. These CIC structures can further form upon exposure to anti-PD-L1 antibodies. CRCSCs, which serve as outer cells in a CIC, also exhibit resistance to natural killer (NK) cells. These findings suggest a novel mechanism by which cancer cells achieve immune evasion via physical cell contact, further driving cancer progression.

Although we observed a much lower frequency of heterotypic CIC structures *in vitro* and *in vivo*, previous studies noted that these structures play a role in cancer progression. In animal bone marrow, heterotypic CIC structures are most commonly observed between neutrophils and megakaryocytes, especially following sublethal irradiation or blood loss 49, 50. Similar neutrophil-in-carcinoma cell structures were observed in the well-differentiated buccal mucosa squamous cancer cell subline H157-H1/2 51. Our study revealed that HCT15-SDCSCs, but not HT29-SDCSCs, generated more heterotypic CIC structures with dHL60 cells than parental CRC cells, and the SDCSCs mainly served as outer cells. These results indicate that the ability to generate a neutrophil-in-carcinoma cell structure is associated with CSC properties in at least a subset of cases. Since protumor neutrophils can be educated and generated by CRCSC-released sEVs 17, neutrophil-in-CRCSC structures may be another way CRCSCs influence the role of neutrophils in the tumor microenvironment. Consistently, the CIC structures identified in CRC FFPE sections were mainly homotypic **(Figure 6D).** The CIC structure frequency was more remarkable in poorly differentiated adenocarcinomas with pleomorphic morphology than those with MMR deficiency or signet ring cell morphology **(Figure S6A-D)**. These results are compatible with the general pathological observation that CIC is a feature of poorly differentiated malignancies with pleomorphic cells.

The formation of CIC structures, specifically homotypic CIC structures, suggests that the two interacting cells have different degrees of deformability. The RHO/RAC1 signaling pathway is the primary determinant of this property. The RHO-ROCK pathway in the inner cell can facilitate actomyosin contraction, enabling entotic invasion into neighboring cells 52. In contrast, oncogenic KRAS mutations can activate RAC1, softening cancer cells and promoting an outer cell fate within a CIC structure 39. In our study, we observed elevated levels of RAC-GTP in both cell lines tested. Specifically, the HCT15 cell line was KRAS mutated. However, silencing RAC1 and restoring MLC2 phosphorylation suppressed neither homotypic CIC formation **(Figure 4D-E and Figure S5E)** nor the outer cell fate of SDCSCs **(Figure 4F and Figure S5F)**, suggesting that RAC1 may be dispensable for CSC-driven CIC formation.

We examined the alternative pathways involved in CIC structure formation and found that lower STMN1 expression in CSCs is associated with CIC structure formation and outer cell fate. In cancers, STMN1 expression is associated with a malignant phenotype and has been proposed as a therapeutic target 53. High STMN1 levels are linked to aggressive phenotypes in breast cancer 54, while its regulation by tumor suppressor miRNAs, such as miRNA-223 and miR-34a 55, 56, underscores its role in tumor progression. However, the functional role of STMN1 is context-dependent. D'Andrea *et al.* demonstrated that STMN1 knockout in mice did not affect p53-dependent or RAS-driven tumorigenesis 57. In prostate cancer cells, low STMN1 expression was observed in highly invasive, EMT-like cells isolated from undifferentiated adenocarcinomas. Williams *et al.* further showed that inhibiting STMN1 in prostate cancer cells accelerated metastasis via p38 activation and TGF-β signaling cooperation 58. We previously reported that STMN1 was decreased in CRCSCSs and that the restoration of STMN1 softened the cytoplasmic stiffness of CRCSCs and increased their invasiveness 41. In this study, we demonstrated that reduced expression of STMN1 in CRCSCs conferred resistance to NK-92MI cells and dictated the outer cell fate in homotypic CIC structures. An important future research direction is how such a phenomenon may be connected to the functions of STMN1 (such as microtubule organization).

Inhibiting ROCK activity has emerged as a promising strategy to increase NK cell cytotoxicity, primarily by restoring PI3K-dependent Akt activation 59. Research has further demonstrated that ROCK inhibition, particularly when combined with agents that induce immunogenic cell death, can significantly increase anticancer immunity by increasing the immunogenicity of cancer cells and enhancing their susceptibility to antitumor immunity 60. Targeting the RHO-ROCK-MLC pathway also suppresses entotic CIC structure formation by reducing actomyosin contractility and cell stiffness 52. Increased MLC2 phosphorylation at Ser19 was observed in HCT15 **(Figure 4B)** and HT29 **(Figure S5B)** parental cells. These findings suggest that inhibiting MLC2 phosphorylation may reduce homotypic CIC structure formation from the perspective of the inner cell. When cells were treated with the ROCK inhibitor Y27632, we observed decreased MLC2 phosphorylation (Ser19) in HCT15 and HT29 parental cells **(Figure S7A)**. The CIC structures **(Figure S7B)** and the outer cell fate of SDCSCs were suppressed **(Figure S7C)** in the presence of ROCK inhibition in the coculture. The administration of Y27632 also modestly increased the sensitivity of parental HCT15 cells to NK92-MI cytotoxicity **(Figure S7D)**. These findings highlight the potential application of ROCK inhibitors in treating CRC, specifically in combination with anti-CSC therapeutics.

Our human tumor specimen study revealed correlations between CIC structures, poor adenocarcinoma differentiation, low STMN1 expression, and poor patient prognosis, indicating the clinical relevance of our current study. Our findings of the associations between CIC structure, anti-PD-L1 antibody exposure, and NK cytotoxicity susceptibility suggest that the CIC structure frequency may be a general prognostic factor and an important marker when considering immune therapy for CRC patients. Mismatch repair-deficient (dMMR) colorectal cancer is known to be the most responsive to ICBT. Although such tumors are often poorly differentiated, with little glandular structure formation, the tumor cells usually form uniform sheets of cells. Our observations revealed that these tumor cells rarely engage in CIC structure formation. In contrast, pleomorphic carcinomas, another type of poorly differentiated adenocarcinoma, frequently exhibited CIC structure formation in our study **(Figure S6D)**, and these carcinomas typically do not show significant responses to ICBT clinically. It is likely that CIC structure formation, with its effect on the TME, is another contributing factor to the immune therapy response independent of the genomic instability generated by mismatch repair deficiency. Investigating cohorts of patients who have received immunotherapy is an important future direction for determining whether CIC structure formation is an independent predictor of immunotherapy response.

## Conclusions

CRCSCs can form homotypic CIC structures with other nonstem-like cancer cells, often playing the role of outer cells. CRCSCs engaged in this activity gain resistance to NK cytotoxicity. This phenomenon can be suppressed by STMN1 overexpression. In human CRC specimens, CIC structure formation is associated with low STMN1 expression, poor tumor differentiation, and an inferior prognosis **(Figure 6N)**.

## Supplementary Material

Supplementary figures and tables, movie legends.

Supplementary movies.

## Abbreviations

cDNA: complementary DNA
CIC: cell-in-cell
CRC: colorectal cancer
CSCs: cancer stem cells
DFS: disease-free survival
dMMR: mismatch repair deficient
DMSO: dimethyl sulfoxide
EDTA: ethylenediaminetetraacetic acid
EMT: epithelial-mesenchymal transition
EV: extracellular vesicle
FFPE: formalin-fixed, paraffin-embedded
GSH: glutathione
GST: glutathione s-transferase
HE: hematoxylin and eosin
HEK: human embryonic kidney
hpf: high-power fields
ICBT: immune checkpoint blockade therapy
IHC: immunohistochemistry
MMR-D: mismatch repair deficiency
MSCs: mesenchymal stem cells
NK: natural killer
PBS: phosphate-buffered saline
PFA: paraformaldehyde
PMA: phorbol 12-myristate 13-acetate
poly-HEMA: poly 2-hydroxyethyl methacrylate
RT‒qPCR: real-time quantitative PCR
SD: standard deviation
SDCSCs: sphere-derived cancer stem cells
sEVs: small extracellular vesicles
STR: short tandem repeat
TME: tumor microenvironment
TRAIL: TNF-related apoptosis-inducing ligand
