# Supplementary material for: Colorectal cancer stem cells develop NK cell resistance via homotypic cell-in-cell structures suppressed by Stathmin1
Source: Theranostics. 2025 Mar 18;15(10):4308–24. doi: 10.7150/thno.110379 (PMC11984389; doi:10.7150/thno.110379)
Supplement: Supplementary file 1 — Supplementary figures and tables, movie legends. [file thnov15p4308s1.pdf]

Supplementary Material

Colorectal cancer stem cells develop NK cell resistance via homotypic cell-in-cell structures suppressed by Stathmin1

Yen-Yu Lin, Hsin-Yi Lan, Hao-Wei Teng, Ya-Pei Wang, Wen-Chun Lin and Wei-Lun Hwang*

***Corresponding author:**

Wei-Lun Hwang, Ph.D.

Department of Biotechnology and Laboratory Science in Medicine, National Yang-Ming University, Taipei, Taiwan. Email: wlhwang@nycu.edu.tw; Phone: 886-2-28267000 ext. 65832; Fax: 886-2-28264092; Postal address: Department of Biotechnology and Laboratory Science in Medicine, National Yang Ming Chiao Tung University; No. 155, Sec. 2, Li-Nong Street, Taipei, 112 Taiwan

This file includes:

Figures S1 to S8

Tables S1 to S4

Movie legends S1 to S6

Supplementary figures and legends

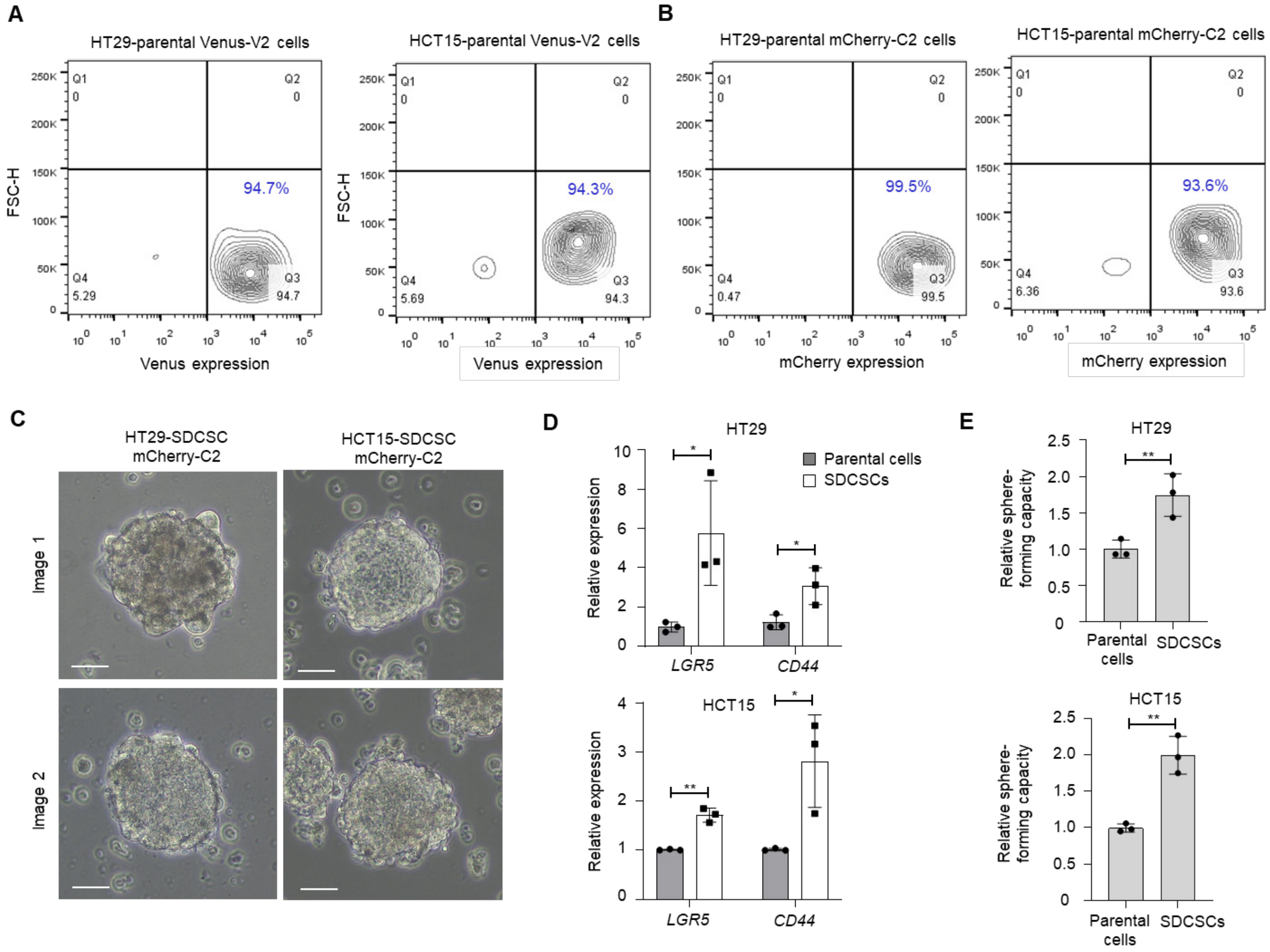

Figure S1: Expansion and characterization of fluorescent protein-carrying CRC cells. (A-B). Percentages of Venus (V2)-positive parental cells (A) and mCherry (C2)-positive parental cells (B) in HT29 cells (left panels) and HCT15 cells (right panels) post cell sorting. The purity is indicated in the Q3 region. **(C).** Representative images of expanded mCherry-carrying HT29-SDCSCs and HCT15-SDCSCs. Scale bar = 50 µm. **(D).** Expression of stemness genes in parental cells (Venus-V2) and SDCSCs (mCherry-C2). Data is represented as means ± sds. *P < 0.05, **P < 0.01 estimated by student's T-test. *N* = 3. **(E)** Histograms show the relative sphere-forming capacity in parental cells (Venus-V2) and SDCSCs (mCherry-C2). Data is represented as means ± sds. **P < 0.01 estimated by student's T-test. *N* = 3.

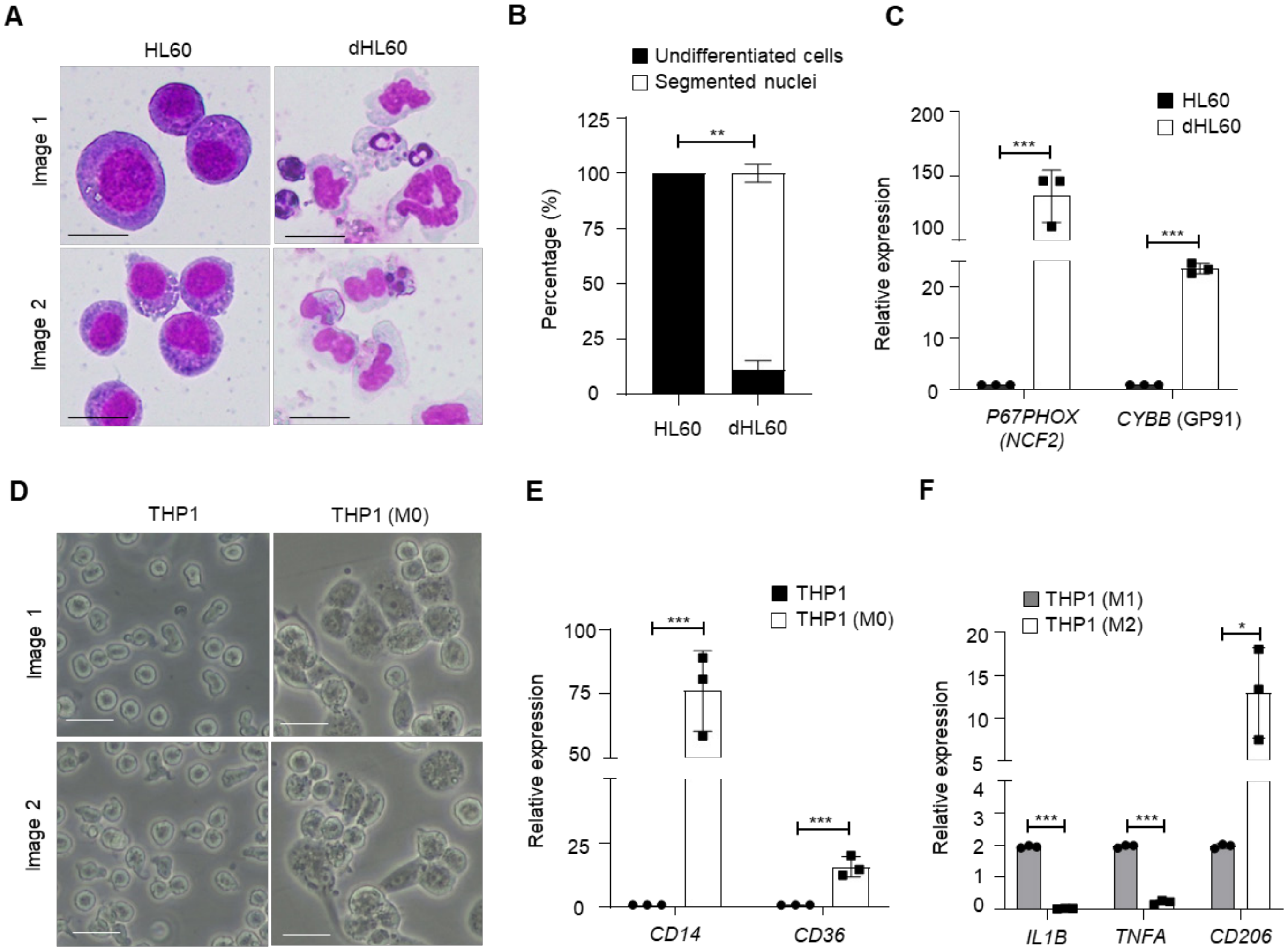

Figure S2: Characterization of differentiated HL60 neutrophil-like cells and differentiated macrophage-like THP1 cells. (A). Representative images of parental HL60 cells and HL60 cells treated with 1.25% DMSO for 8 days for neutrophil differentiation. Cells were collected and spun onto glass slides for Liu's stain. dHL60, differentiated HL60 neutrophil-like cells. Scale bar = 20 μm. Two representative images from three independent experiments. **(B)**. A histogram shows the percentage of undifferentiated blast cells and differentiated cells with segmented nuclei. Data is represented as means ± sems. **P < 0.01 was estimated by two-way ANOVA followed by Tukey's post hoc test. **(C).** A histogram shows the expression of *P67PHOX* (NCF2) and *CYBB* (GP91) in indicated cells. Data is represented as means ± sds. ***P < 0.001 estimated by student's T-test. *N* = 3. **(D).** Representative images show the morphology of resting macrophage-like THP1 cells (THP1-M0). THP1 cells were treated with PMA for one day and maintained in a complete RPMI ATCC formulation medium supplemented for another day. Scale bar = 20 μm. Two representative images from three independent experiments. **(E)**. A histogram shows the expression of *CD14* and *CD36* in indicated cells. Data is represented as means ± sds. ***P < 0.001 estimated by student's T-test. *N* = 3. **(F).** A histogram shows the expression of *IL1B*, *TNFA*, and *CD206* in indicated cells. THP1-M0 cells were treated with IFN-γ and LPS one day for M1-type macrophage differentiation (THP1-M1) or IL-4 and IL-13 for M2-type

macrophage differentiation (THP1-M2). Data is represented as means ± sds. *P<0.05, ***P<0.001 estimated by student's T-test. $N = 3$.

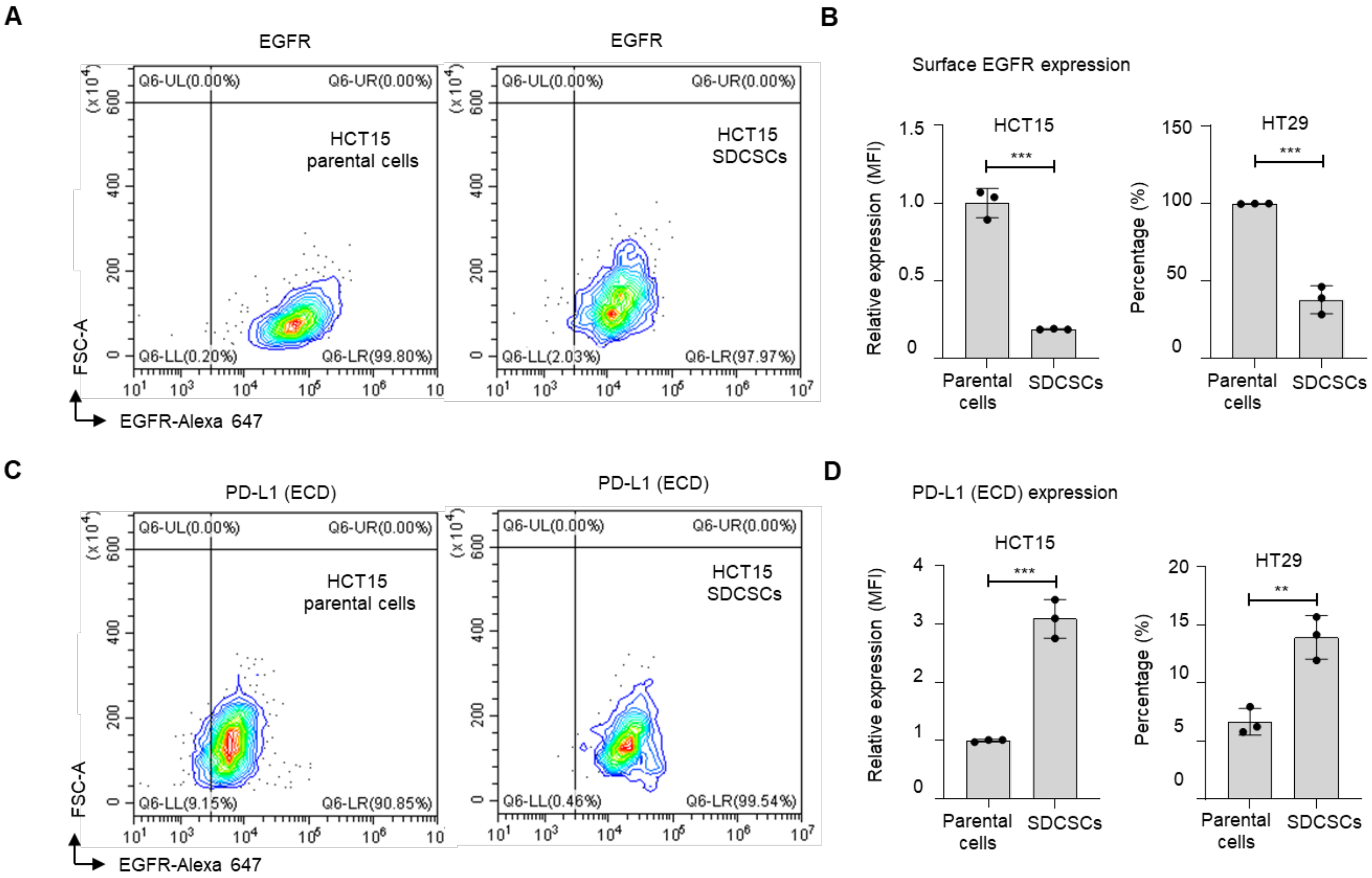

Figure S3: Expression of membranous EGFR and PD-L1 on CRC cells. (A). Representative density plots show the epidermal growth factor receptor (EGFR) expression. FSC, forward scatter. **(B).** Histograms show the expression of EGFR in indicated CRC cells. MFI, mean fluorescent intensity. Data is represented as means ± sds. ***P < 0.001 estimated by student's T-test. N=3. **(C).** Representative density plots of the extracellular domain of programmed cell death-Ligand 1 (PD-L1 ECD). FSC, forward scatter. **(D).** Histograms show the expression of PD-L1 (ECD) in indicated CRC cells. MFI, mean fluorescent intensity. Data is represented as means ± sds. **P < 0.01, ***P < 0.001 estimated by student's T-test. $N = 3$.

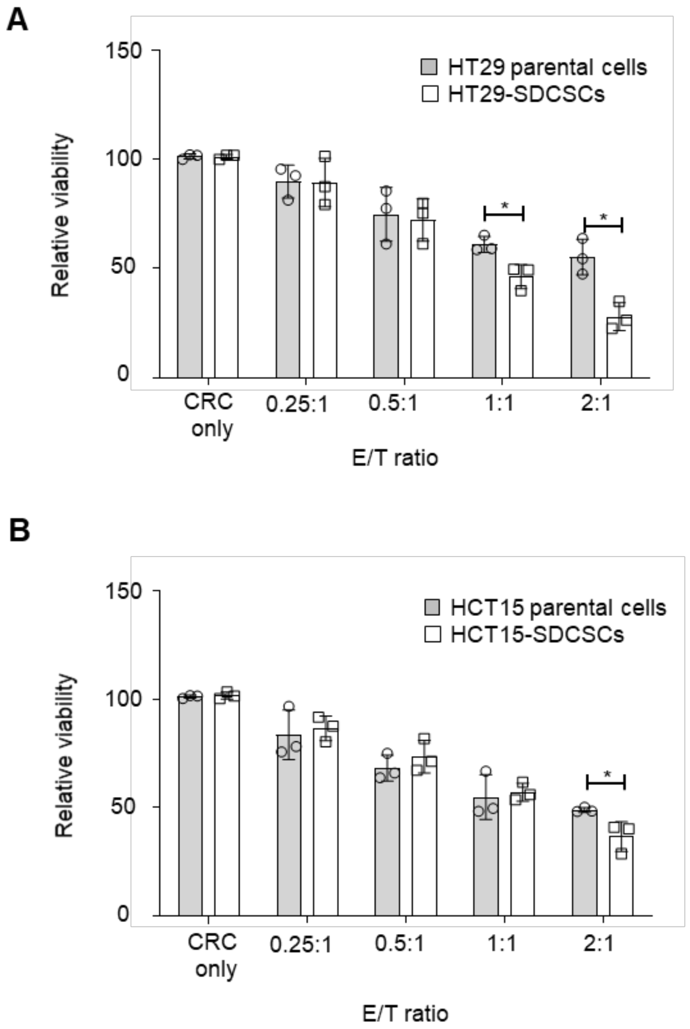

Figure S4: HCT15-SDCSCs and HT29-SDCSCs are sensitive to NK-92MI killing. (A-B). Histograms show the relative viability of HT29 cells **(A)** and HCT15 cells **(B)** in the presence of NK-92MI under the indicated effector-to-target cell ratio (ET ratio). Data is represented as means ± sds. *P < 0.05 estimated by student's T-test. *N* = 3.

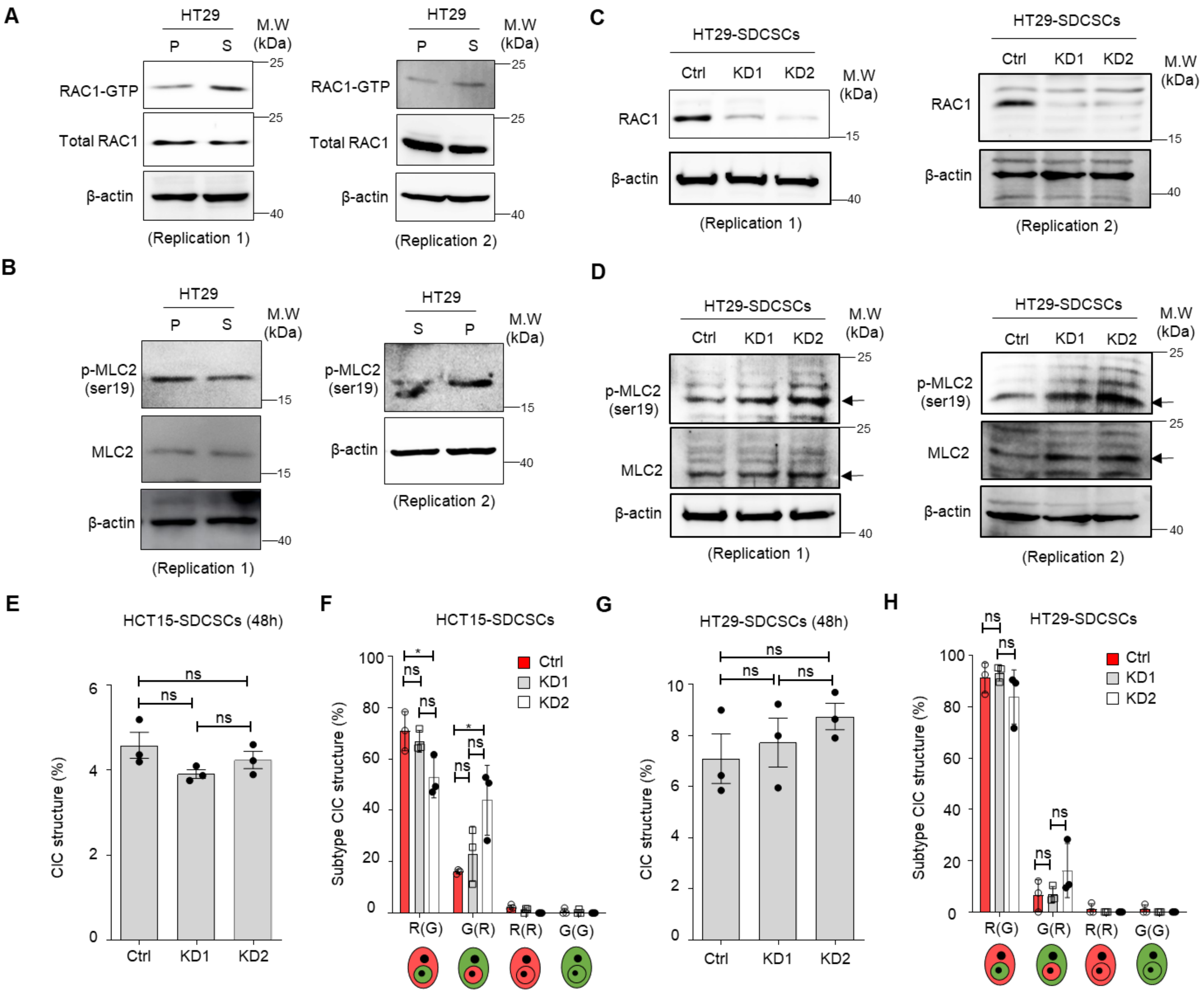

Figure S5: Knocking down *RAC1* in HT29-SDCSCs does not suppress the CIC frequency and the outer cell fate. (A). Representative images show the RAC1-GTP and total RAC1 expression in parental cells (P) and SDCSCs (S). M.W., molecule weight. **(B).** Representative images show the phosphorylated MLC2 (Ser19) and total MLC2 expression in parental cells (P) and SDCSCs (S). **(C).** Representative images show total RAC1 expression HT29-SDCSCs receiving scramble control shRNA (pLKO.1) or shRNAs targeting *RAC1* (KD1 and KD2). **(D).** Representative images show the phosphorylated MLC2 (Ser19) and total MLC2 expression in indicated cells. **(E).** Percentages of CIC structures generated by Venus-labeled parental cells indicated that mCherry-marked SDCSCs (pLKO.1 control, KD1, and KD2) were in an RPMI basal medium for 48 hours. Data is represented as means ± sems. ns, non-significance estimated by student's T-test. $N = 3$. **(F).** Percentages of inner/ outer cell fates of mCherry-labeled SDSCCs carrying pLKO.1 control or shRNAs targeting RAC1 (KD1 or KD2) and Venus-labelled parental CRC cells in CIC structures 48 hours post cell seeding. There were 112 (pLKO.1), 120 (KD1), and 128 (KD2) CIC

structures counted when co-culturing HCT15 parental cells (V2), and indicated HCT15-SDCSCs (C2). There were 92 (pLKO.1), 90 (KD1), and 128 (KD2) CIC structures counted when co-culturing HT29 parental cells (V2) and indicated HT29-SDCSCs (C2). R(G), CIC structures with outer mCherry-SDCSCs (Red, C2) and inner parental CRC cells (Green, V2). Data is represented as means ± sems. *P < 0.05. ns, non-significance estimated by student's T-test. $N = 3$.

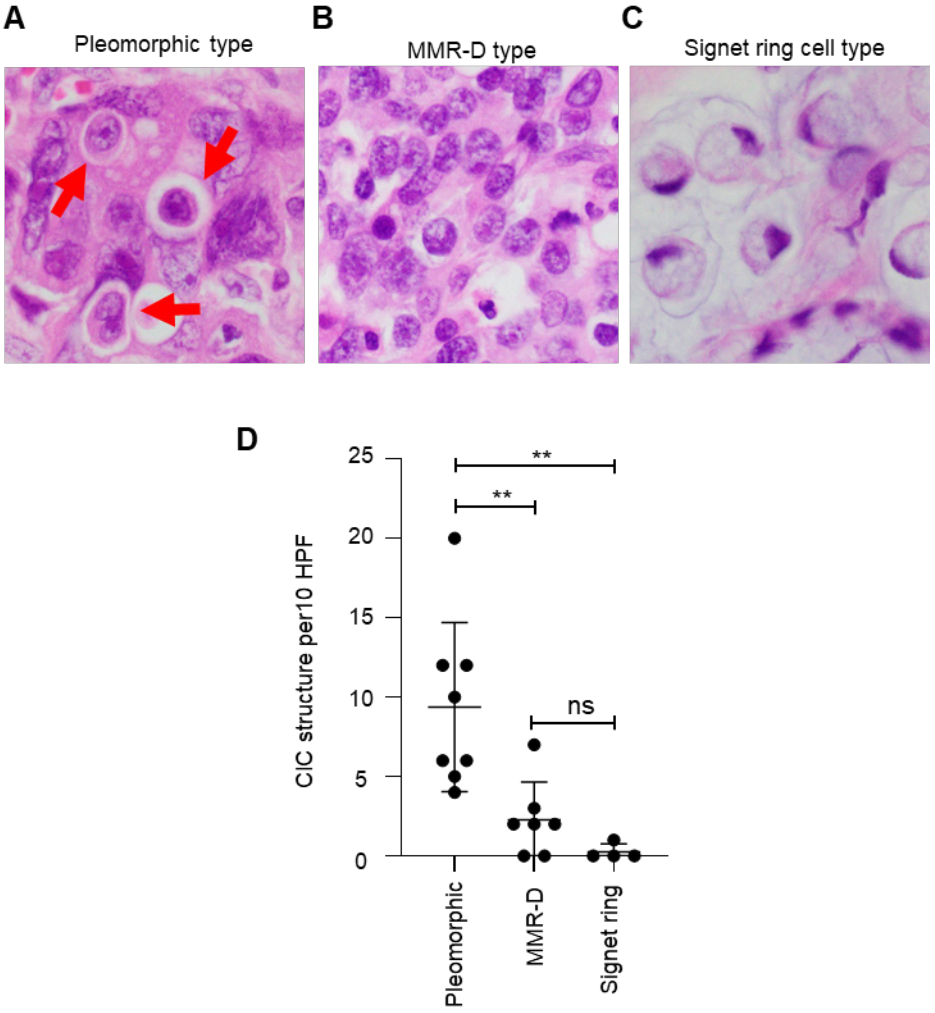

Figure S6: Increased CIC structures in undifferentiated CRC specimens with pleomorphic histological features. (A-C). Representative images of undifferentiated CRC tissues. In poorly differentiated cases, pleomorphic tumor cells (A), mismatch repair protein-deficient (MMR-D) (B), and signet ring cells (C) were noted. Arrows, CIC structures. **(D)**. A histogram quantifying CIC structures in undifferentiated CRC tissue with different histological features. Data is represented as means ± sds. **P < 0.01. ns, non-significance estimated by Mann-Whitney test. MMR-D: mismatch repair protein-deficient. hpf, high power field.

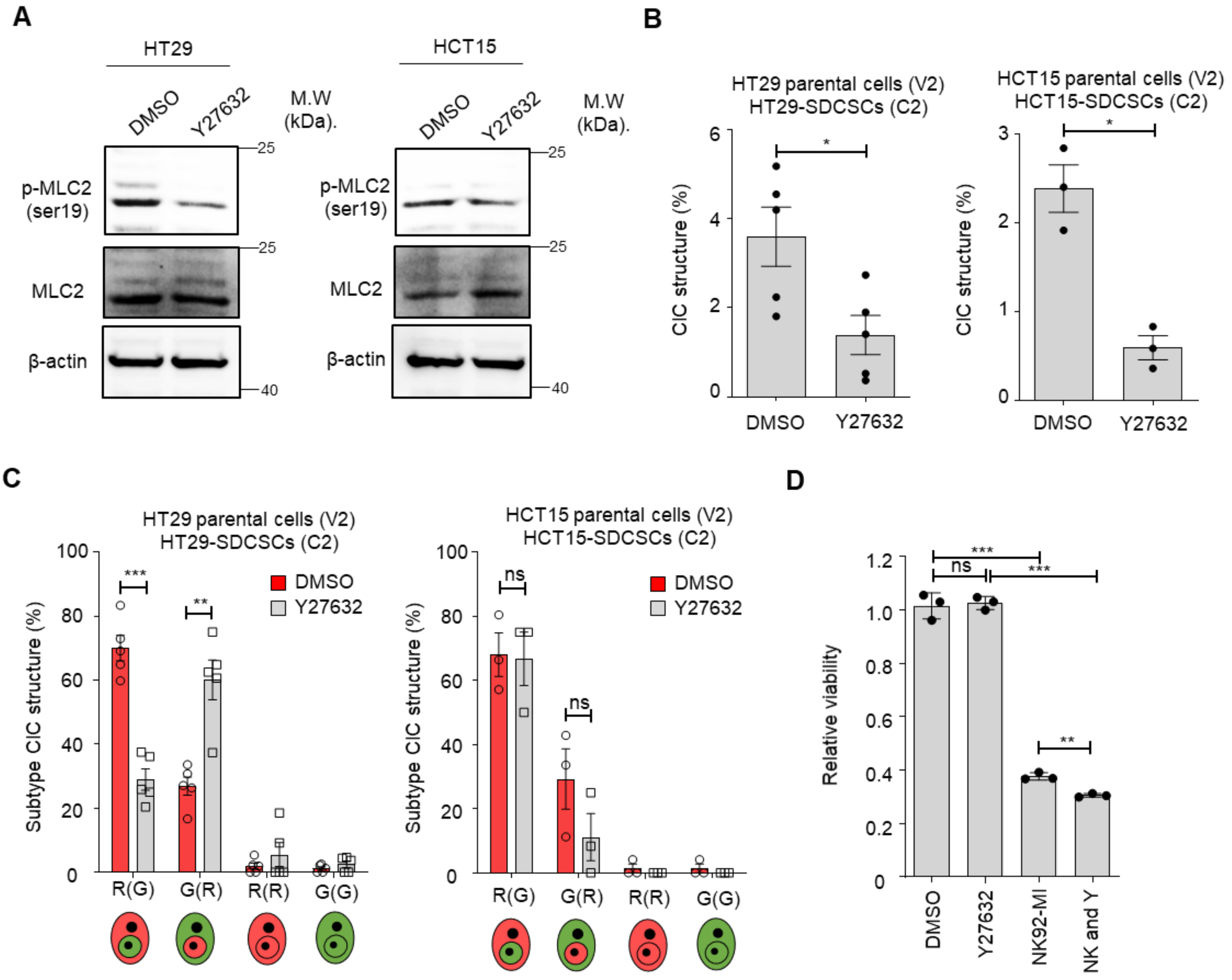

Figure S7: Inhibition of ROCK activity suppresses the homotypic CIC structure formation and sensitizes parental CRC cells to NK92-MI killing. (A). Representative images show the phosphorylated MLC2 (Ser19) and total MLC2 expression in parental CRC cells treated with vehicle control (DMSO) or a ROCK inhibitor Y2732 (50 μM) in basal RPMI-1640 medium for 24 hours. M.W., molecule weight. **(B).** Percentage of CIC structures generated by Venus-labeled parental cells and mCherry-marked SDCSCs in the presence of Y27632 in RPMI basal medium for 24 hours. Data is represented as means ± sems. *$P < 0.05$ estimated by student's T-test. $N = 5$ in HT29 groups and $N = 3$ in HCT15 groups. **(C).** Percentages of CIC structure subtypes of Venus-labeled parental cells and mCherry-marked SDCSCs in the presence of vehicle control (DMSO) or Y27632 in RPMI basal medium for 24 hours. There were 254 (DMSO), and 106 (Y27632) CIC structures counted when co-culturing HT29 parental cells (V2) and HT29-SDCSCs (C2). There were 59 (DMSO), and 18 (Y27632) CIC structures counted when co-culturing HCT15 parental cells (V2) and HCT15-SDCSCs (C2). R(G), CIC structures with outer mCherry-SDCSCs (Red, C2) and inner parental CRC cells (Green, V2). Data is

represented as means ± sems. *P < 0.05. ns, non-significance estimated by student's T-test. *N* = 5 in HT29 groups and *N* = 3 in HCT15 groups. **(D).** Histograms show the relative viability of parental HCT15 cells (V2) pre-treated with Y27632 (50 μM) 24 hours before NK-92MI administration at the effector-to-target cell ratio (0.5:1). The Y27632 was washed away before NK92-MI treatment. Data is represented as means ± sds. **P < 0.01, ***P < 0.001. ns, non-significance estimated by student's T-test. *N* = 3.

Figure 4A, replication 1

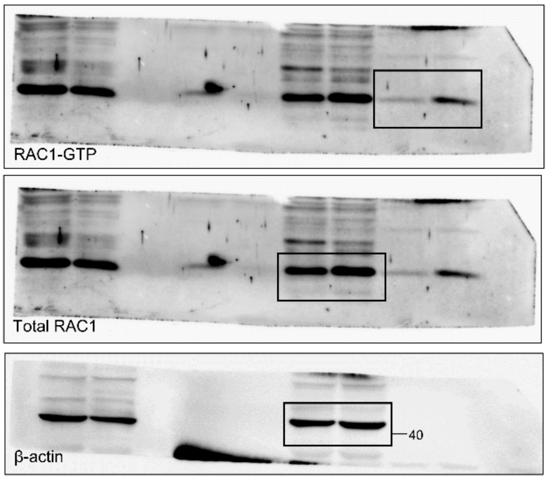

Figure 4A, replication 2

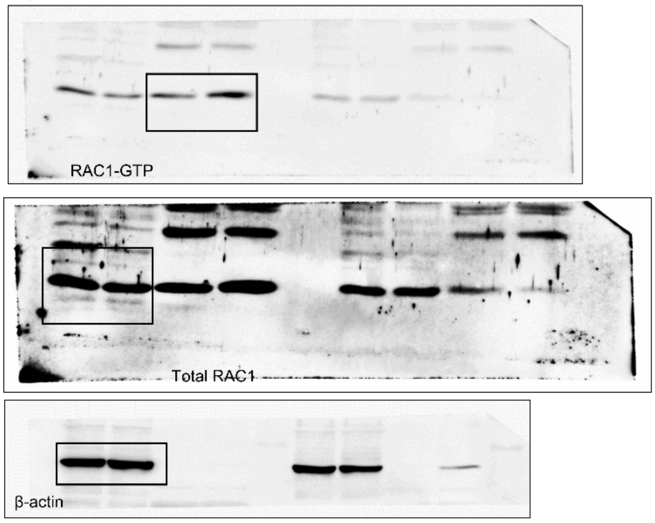

Figure 4B, replication 1 and 2

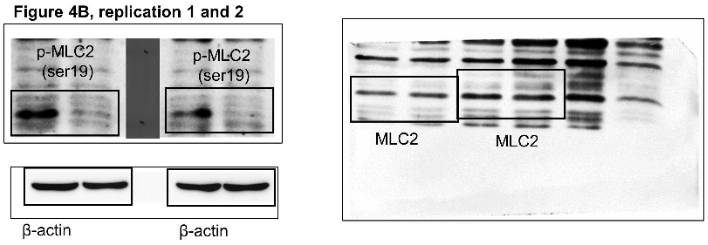

Figure 4C, replication 1 and 2

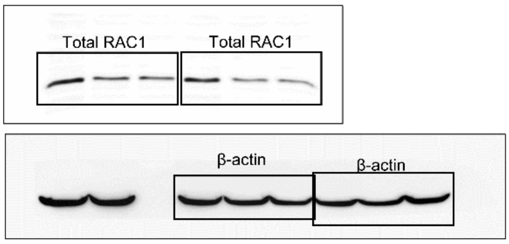

Figure 4D, replication 1

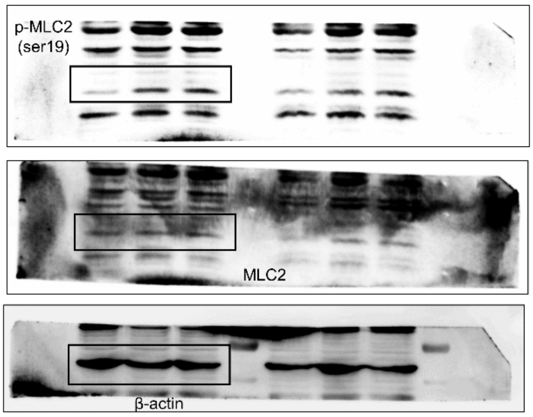

Figure 4D, replication 2

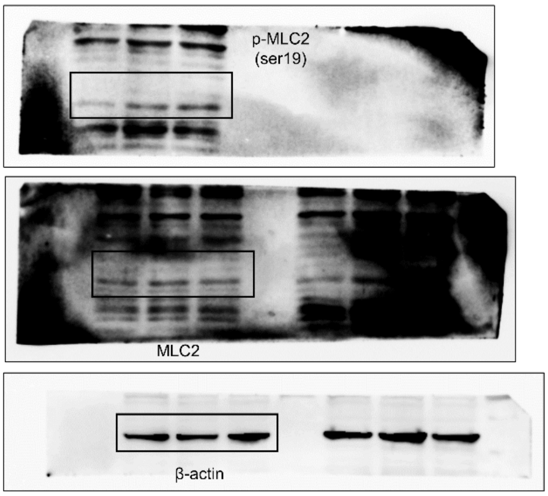

Figure 5A, replication 1 and 2

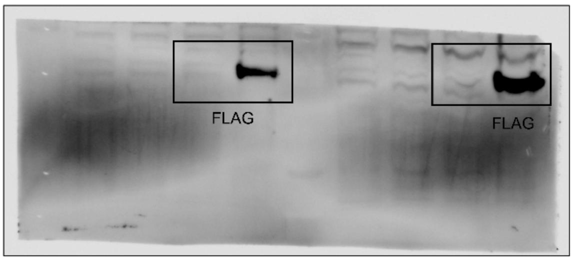

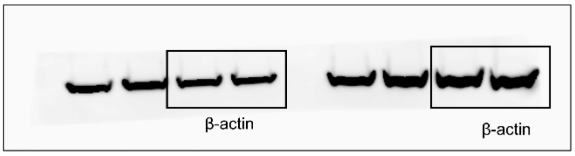

Figure 5B, replication 1

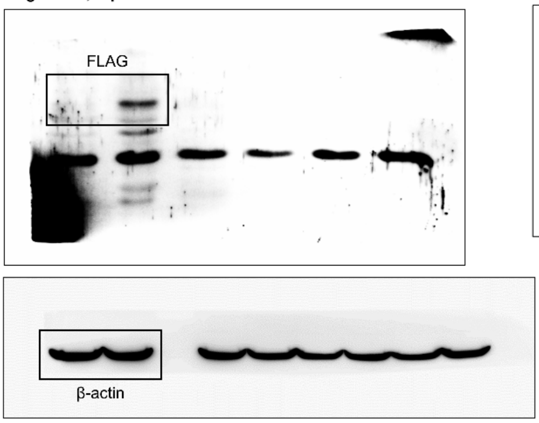

Figure 5B, replication 2

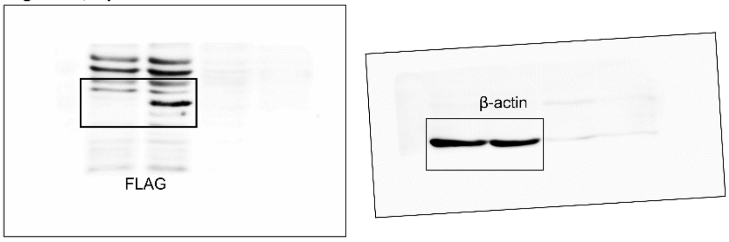

Figure S5B, replication 1

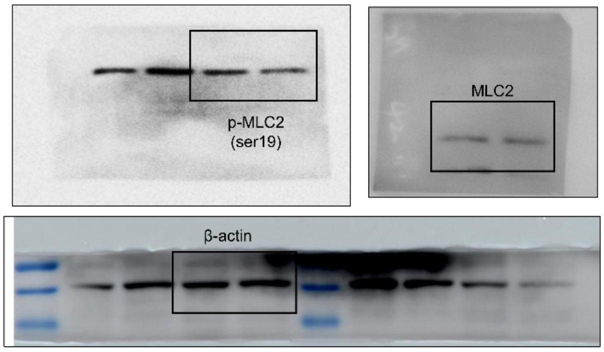

Figure S5A, replication 1

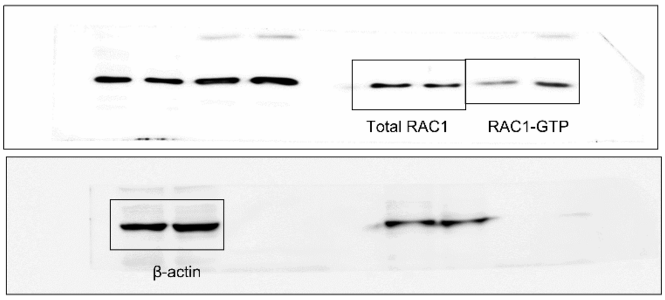

Figure S5C, replication 1

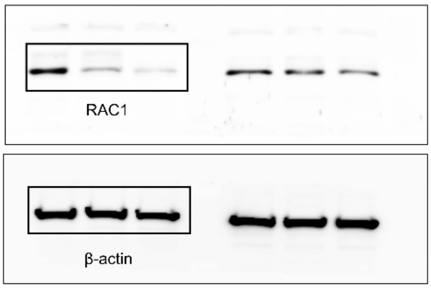

Figure S5A, replication 2

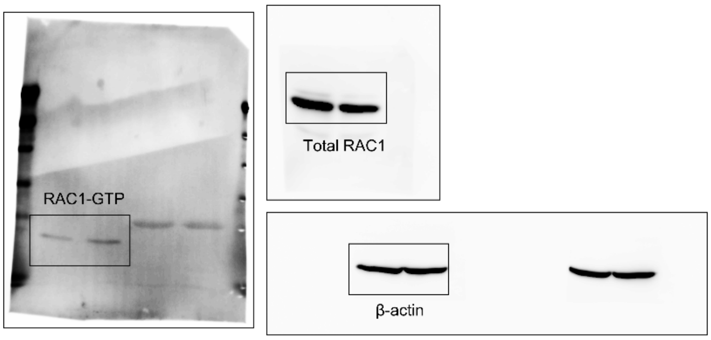

Figure S5C, replication 2

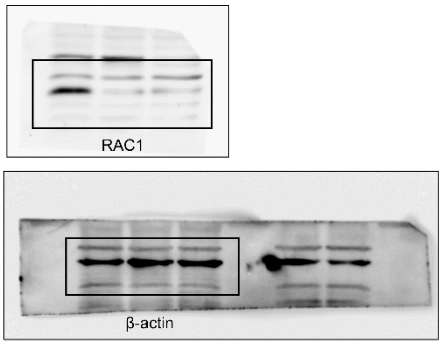

Figure S5B, replication 2

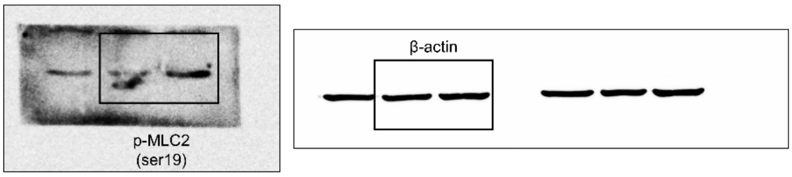

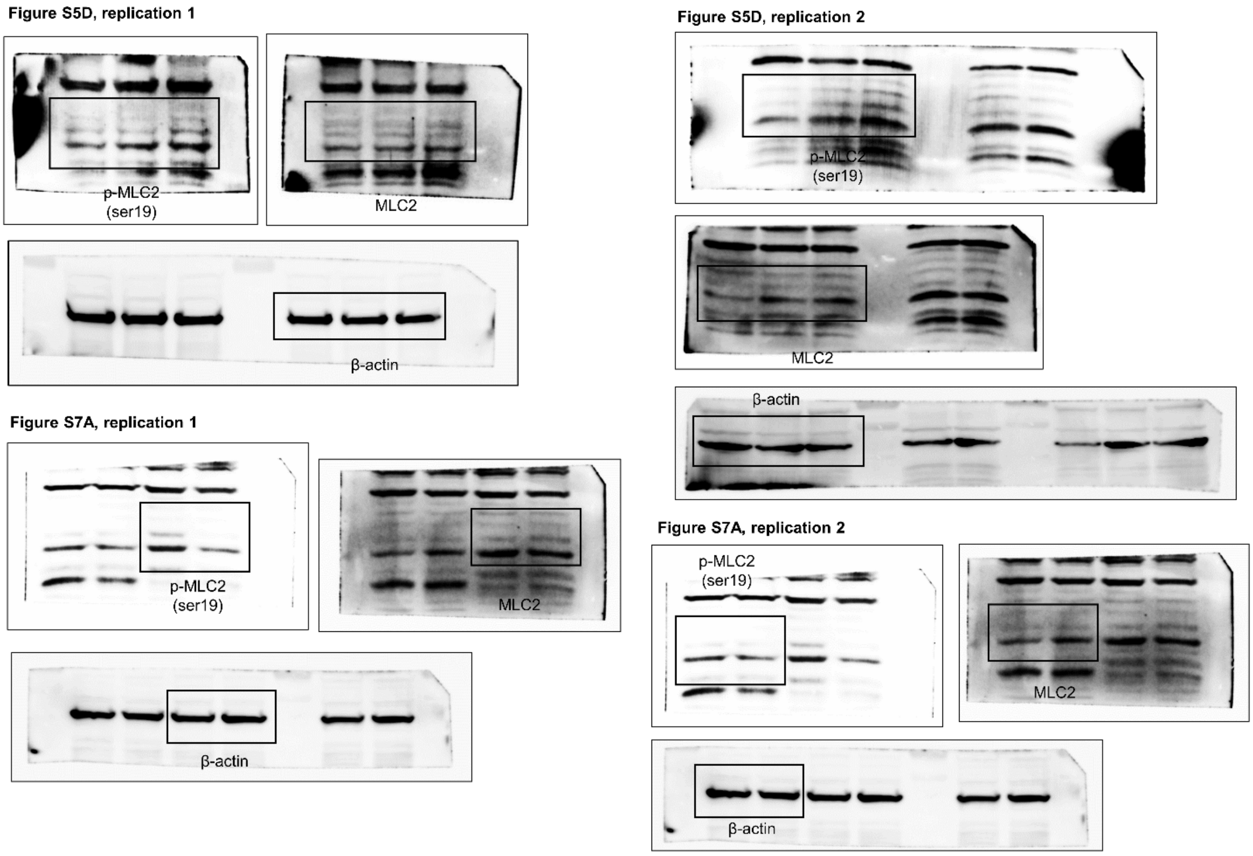

Figure S8: Uncropped western blots. The original blots in the indicated figures are shown here.

Supplementary tables

Supplementary Table 1: Clinical and pathological information of CRC patients

Pathology Group	Poorly Differentiated (n=19)	Well- to moderately differentiated (n=19)
Mean Age (Range)	64 (37-86)	63 (32-92)
Sex (Male: Female)	12:7	10:9
Stage Group		
I	1	0
IIA	2	8
IIIA	1	0
IIIB	3	6
IIIC	4	5
IVA	5	0
IVC	3	0
Mismatch Repair Protein Expression Status		
dMMR	7	7
pMMR	12	12

dMMR: mismatch repair protein deficient, pMMR: mismatch repair protein proficient

Supplementary Table 2: Primer sequences.

Gene Symbol	Forward Primer	Reverse Primer
CD44	CCA GAT GGA GAA AGC TCT GA	GTC ATA CTG GGA GGT GTT TGG
LGR5	TGT TGG GAG ATC TGC TTT C	CAG ACG GTT TGA GGA AGA GA
P67(PHOX)	GTC AGT TGC CAA AAG GTG GG	GAA GAC AGG TTG GAG CGT CT
CYBB (GP91)	GGG AAC TGG GCT GTG AAT GA	CCA GTG CTG ACC CAA GAA GT
CD14	CTGGAACAGGTGCCTAAAGGAC	GTCCAGTGTCAGGTTATCCACC
CD36	CAGGTCAACCTATTGGTCAAGCC	GCCTTCTCATCACCAATGGTCC
IL1B	CCA CAG ACC TTC CAG GAG AAT G	GTG CAG TTC AGT GAT CGT ACA GG
TNFA	CTC TTC TGC CTG CTG CAC TTT G	ATG GGC TAC AGG CTT GTC ACT C
CD206	AGCCAACACCAGCTCCTCAAGA	CAAAACGCTCGCGCATTGTCCA
GAPDH	AAG GTC GGA GTC AAC GGA TTT G	CCA TGG GTG GAA TCA TAT TGG AA

Supplementary Table 3: Antibody list.

Antibody Name	Source	Identifier	Application
Mouse Alexa Fluor 647 anti-human IgG Fc antibody	BioLegend	409320	FC (1:100)
Mouse Alexa Fluor 647-conjugated anti-human EGFR antibody (clone AY13)	BioLegend	352918	FC (1:100)
Rabbit Alexa Fluor 647-conjugated humanPD-L1-extracellular domain (clone 28-8)	Abcam	ab209960	FC (1:100)
Mouse IgG2b control (clone MPC-11)	Bioxcell	BE0086	Neutralizing (200 μg/ml)
Mouse anti-human PD-L1 (B7-H1) antibody (clone 29E.2A3)	Bioxcell	BE0285	Neutralizing (200 μg/ml)
Chimeric human Cetuximab antibody	Merck	Erbitux	Neutralizing (200 μg/ml)
Goat Alexa Fluor 488-conjugated anti-mouse IgG	Abcam	ab150117	IFA (1:200)
Goat Alexa Fluor 546-conjugated anti-rabbit IgG	Thermo Fisher Scientific	A-11035	IFA (1:1000)
Rabbit anti-human STMN1 (clone D1Y5A)	Cell Signaling	13655	IHC (1:500)
Rabbit anti-human CD45 (clone D9M8I)	Cell Signaling	13917	IFA (1:400)
Mouse anti-human cytokeratin (clone AE1/AE3)	Agilent	M3515	IFA (1:600)
Rabbit anti-human RAC1 antibody	Invitrogen	PA1-091	WB (1:1000)
Mouse anti-FLAG M2 antibody	Sigma-Aldrich	F1804	WB (1:1000)
Rabbit anti-MLC2 antibody	Cell Signaling	3672	WB (1:1000)
Mouse anti-human phosphorylated MLC2 (S19) antibody	Cell Signaling	3675	WB (1:500)
Goat anti-mouse IgG-HRP	Genetex	GTX213111-01	WB (1:5000)
Goat anti-rabbit IgG-HRP	Genetex	GTX26721	WB (1:5000)
Mouse anti-β-actin antibody	Proteintech	66009-1	WB (1:5000)

FC, flow cytometry; IFA, immunofluorescent assay; IHC, immunohistochemistry; WB, western blot

Supplementary Table 4: List for reagents and chemicals used in this study.

Reagents and chemicals	Source	Identifier
Liu's staining solution	Tonyar biotech.Inc.	B.1850
Phorbol 12-myristate 13-acetate (PMA)	Sigma-Aldrich	P1585
Recombinant IFN-γ	PeproTech	300-02
LPS	Sigma-Aldrich	L-8274
Recombinant IL-4	PeproTech	200-04
Recombinant IL-13	PeproTech	200-13
RPMI 1640 medium (ATCC modification)	Thermo Fisher Scientific	A1049101
Recombinant EGF	PeproTech	AF-100-15
Recombinant bFGF	PeproTech	AF-100-18B
N2	Thermo Fisher Scientific	17502-048
Mycoexpert	Capricorn Scientific	MYX-B
shRNA RAC1	RNA Technology Platform and Gene Manipulation Core Facility (RNAi core)	TRCN0000004871
shRNA RAC1	RNA Technology Platform and Gene Manipulation Core Facility (RNAi core)	TRCN0000004873
lipophilic tracer DiD	Thermo Fisher Scientific	D7757
Dimethyl sulfoxide (DMSO) for cell culture	Fisher BioReagents	BP231-100
2-Mercaptoethanol (2ME)	Gibco	21985-023
Antibody dilution buffer	Ventana	ADB250
Anti-immunoglobulins	BioGenex	QP900-9LE (C)
BCA protein assay kit	Thermo Fisher Scientific	23227
Bovine serum albumin (BSA)	Bioshop	ALB001
Cell culture lysis 5x reagent	Promega	E153A
Glass bottom 3.5cm dish (4 compartments)	Greiner Bio-One	627870
Citric acid	Honeywell	27109
DAB solution	Epredia	TA-060-QHDX
DAPI	Sigma-Aldrich	SI-F6057
Dimethyl sulfoxide (DMSO) for MTT assay	Scharlau	SU01551000
DMEM medium	Gibco	11965-084
Ethanol	Honeywell	32221
Ethylenediaminetetraacetic acid (EDTA)	J.T.Baker	2589937
Fetal bovine serum (FBS)	Gibco	26140079
Fetal bovine serum (FBS)	Hyclone	SH30084.03

Folic acid	Sigma-Aldrich	F8758
Glutathione (GSH) sepharose	Cytiva	17513201
Horse serum	Gibco	16050-122
Inositol	Sigma-Aldrich	I7508
Kaiser's glycerol gelatin mounting medium	Millipore	1.09242.0100
LeGO-C2 (mCherry)	Addgene	27339
LeGO-V2 (Venus)	Addgene	27340
Mayer's hemalum solution	Sigma-Aldrich	1.09249.0500
MEM alpha medium	Gibco	12561-049
Paraformaldehyde (PFA)	Sigma-Aldrich	P6148
Penicillin/ streptomycin	Gibco	15140-122
Phosphatase inhibitor	ThermoFisher	P5726
Phosphate-buffered saline (PBS)	Bioman	PBS101000
pLenti-MYC-DDK.STMN1 plasmid	Origene	RC205073L3
pLenti-Vector plasmid	Origene	PS100092
pMDLg/pRRE plasmid	Addgene	12251
Poly (2-hydroxyethyl methacrylate), polyhema	Sigma-Aldrich	P3932
Polybrene	Sigma-Aldrich	H9268
Protease inhibitor	ThermoFisher	88666
pRSV-Rev plasmid	Addgene	12253
RPMI 1640 medium	Gibco	11875-085
Streptavidin peroxidase	BioGenex	QP900-9LE (B)
SYBR green master mix	ThermoFisher	4385712
Thiazolyl blue tetrazolium bromide reagent	Sigma-Aldrich	M5655
T-pro NTR III transfection reagent	T-pro biotechnology	JT97-N006M
Triton X-100	Sigma-Aldrich	9002-93-1
Y-27632	Cell Signaling Technology	13624S

Supplementary movie legends

Supplementary movie 1. Time-lapse imaging shows the formation of a CIC structure. Red cell, mcherry-carrying HT29-SDCSCs (C2); green cell, Venus-labeled parental HT29 cells (V2).

Supplementary movies 2-5. Time-lapse imaging shows the cell fate of parental HT29-V2 cells in HT29-SDCSC-C2-derived CIC structures. Red cell, mcherry-carrying HT29-SDCSCs (C2); green cell, Venus-labeled parental HT29 cells (V2). Maintain, the CIC structure is unchanged through the time-lapse imaging **(Movie S2)**; Escape, the release of inner cells **(Movie S3)**; Inner dead, inner cell is dead in a CIC structure **(Movie S4)**; Inner proliferation, the inner cell divides within a CIC structure **(Movie S5)**.

Supplementary movie 6. Three-dimensional (3D) image movie of a representative CIC structure. The image shows complete encirclement of the inner cell by the outer cell and the formation of a vacuolar space around the inner cell. Green, fluorescent lipophilic tracer DiD; red, DAPI (pseudocolor in red).
